# A Localized Materials‐Based Strategy to Non‐Virally Deliver Chondroitinase ABC mRNA Improves Hindlimb Function in a Rat Spinal Cord Injury Model

**DOI:** 10.1002/adhm.202200206

**Published:** 2022-08-25

**Authors:** Andrew S. Khalil, Daniel Hellenbrand, Kaitlyn Reichl, Jennifer Umhoefer, Mallory Filipp, Joshua Choe, Amgad Hanna, William L. Murphy

**Affiliations:** ^1^ Department of Biomedical Engineering University of Wisconsin‐Madison Madison WI 53705 USA; ^2^ Department of Orthopedics and Rehabilitation University of Wisconsin‐Madison Madison WI 53705 USA; ^3^ Department of Neurosurgery University of Wisconsin‐Madison School of Medicine and Public Health Madison WI 53705 USA; ^4^ Department of Biology University of Wisconsin‐Madison Madison WI 53705 USA; ^5^ Medical Scientist Training Program University of Wisconsin‐Madison School of Medicine and Public Health Madison WI 53705 USA; ^6^ Department of Materials Science and Engineering University of Wisconsin‐Madison Madison WI 53705 USA; ^7^ Forward BIO Institute University of Wisconsin‐Madison Madison WI 53705 USA; ^8^ Present address: Whitehead Institute for Biomedical Research Cambridge MA 02142 USA; ^9^ Present address: The Wyss Institute for Biologically Inspired Engineering Boston MA 02115 USA; ^10^ Present address: Virginia Commonwealth University School of Medicine Richmond VA 23298 USA; ^11^ Present address: Biomedical Sciences Program University of California San Francisco CA 94143 USA; ^12^ Present address: Driskill Graduate Program Northwestern University Feinberg School of Medicine Chicago IL 60611 USA

**Keywords:** biomaterials, messenger RNA delivery, non‐viral gene therapy, regenerative medicines, spinal cord injury

## Abstract

Spinal cord injury often results in devastating consequences for those afflicted, with very few therapeutic options. A central element of spinal cord injuries is astrogliosis, which forms a glial scar that inhibits neuronal regeneration post‐injury. Chondroitinase ABC (ChABC) is an enzyme capable of degrading chondroitin sulfate proteoglycan (CSPG), the predominant extracellular matrix component of the glial scar. However, poor protein stability remains a challenge in its therapeutic use. Messenger RNA (mRNA) delivery is an emerging gene therapy technology for in vivo production of difficult‐to‐produce therapeutic proteins. Here, mineral‐coated microparticles as an efficient, non‐viral mRNA delivery vehicles to produce exogenous ChABC in situ within a spinal cord lesion are used. ChABC production reduces the deposition of CSPGs in an in vitro model of astrogliosis, and direct injection of these microparticles within a glial scar forces local overexpression of ChABC and improves recovery of motor function seven weeks post‐injury.

## Graphical abstract

A nonviral, biomaterials‐based, and single‐dose messenger RNA (mRNA) therapy improves motor function recovery in an injured rat spinal cord after glial scar formation, unlike previous studies requiring repeated enzyme injections or immediate treatment before scar formation. As the materials and the mRNA can be readily redesigned to encode other therapeutic proteins, this approach may have applications in other disease locations.

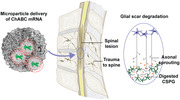

## 1 Introduction

Spinal cord injury (SCI) occurs most frequently as a result of accidental trauma causing fracture and/or dislocation of the spine, puncture or severing of the spinal cord.^[^ ^1^ ^]^ The initial insult breaks down the blood‐spinal cord barrier resulting in ischemia, oxidative damage, edema, and glutamate excitotoxicity.^[^ ^2^, ^3^ ^]^ This sets off a secondary injury cascade in which there is extensive infiltration of immune cells leading to additional cell death and spinal cord damage.^[^ ^2^, ^3^, ^4^, ^5^ ^]^ Resident astrocytes migrate to the injury, proliferate, and upregulate the expression of glial fibrillary acidic protein (GFAP) and chondroitin sulfate proteoglycan (CSPG).^[^ ^1^, ^6^, ^7^, ^8^ ^]^ This reactive astrocytic event creates a “glial scar” that encases the injured area and serves as a permanent chemical and physical barrier that inhibits axonal regeneration.^[^ ^9^, ^10^, ^11^ ^]^ SCI often results in permanent paralysis in regions of the body innervated caudal to where the SCI occurred, with the final outcome generally being paraplegia (paralysis of both legs) or quadriplegia (paralysis of both legs and arms).

**Figure 1:**
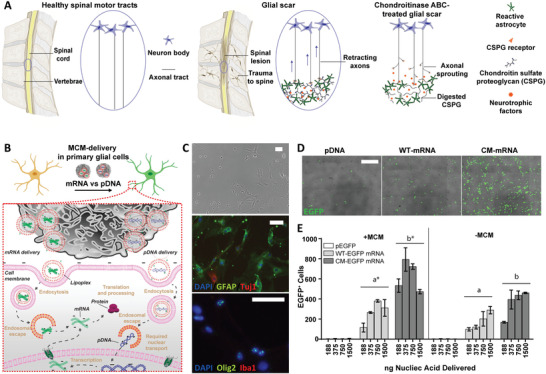
Delivery of both MCMs and mRNA improves transfection efficiency in primary glial cells. A) Schematic illustrating how spinal contusions (left) results in glial scar (middle) and how delivery of chondroitinase ABC degrades the scar and potentially restores function through elimination of the physical barrier and release of sequestered neurotrophic factors. B) Schematic illustrating the differences between pDNA and mRNA delivery. mRNA delivery results in protein production without requiring entry into the cell nucleus. C) Representative images of immunohistochemistry identifying populations of glial cells. scale bar = 50 µm D) Merged phase and green fluorescence micrographs of primary adult glial cells isolated from the rat spinal cord transfected with pDNA, WT‐mRNA and CM‐mRNA using MCMs. Scale bar = 500 µm E) Comparison of transfection yields (EGFP+ cells) between pDNA, WT‐mRNA and CM‐mRNA, with and without MCMs. *N* = 3 a versus a*, and b versus b* represent significant differences in means, mean + SD *p* < 0.05 by two‐way ANOVA with Tukey's post hoc analysis.

While the rate of SCI is low relative to other healthcare concerns (906 per million people in the USA),^[^ ^12^, ^13^ ^]^ the severity of the outcomes, the incident of co‐morbidities,^[^ ^14^ ^]^ and the dearth of any treatment options beyond minimal palliative care^[^ ^15^ ^]^ have motivated attempts to improve outcomes.^[^ ^16^, ^17^, ^18^, ^19^ ^]^ A previously explored method for resolving the glial scar involves administering an enzyme capable of degrading the inhibitory CSPGs,^[^ ^18^, ^19^ ^]^ such as chondroitinase ABC (ChABC). ChABC is a bacterial enzyme that efficiently degrades the Chondroitin sulfate A, B, and C side chains of CSPGs.^[^ ^20^ ^]^ Treatment of glial scar tissue^[^ ^21^, ^22^ ^]^ with this enzyme can result in CSPG degradation^[^ ^11^, ^22^, ^23^ ^]^ (**Figure** **1**A). It has also been shown that digesting these CSPGs within the perineuronal nets promotes axon sprouting and regeneration.^[^ ^24^, ^25^ ^]^ However, ChABC is rapidly denatured at physiological temperatures,^[^ ^26^ ^]^ and its ability to degrade CSPGs that exist or are later deposited in the glial scar is limited by its poor stability.

Previous studies have used sustained delivery to address the challenges associated with ChABC instability. For example, Lee et al. demonstrated that incubation of ChABC protein with the osmolytic sugar trehalose resulted in improved thermal stability of ChABC, and administering ChABC with trehalose resulted in efficient CSPG reduction in an in vivo murine glial scar.^[^ ^27^ ^]^ However, trehalose is a bioactive compound known to induce autophagy,^[^ ^28^ ^]^ and so its use as a component of ChABC therapy could lead to undesirable side effects. In an alternative approach, a large bolus injection of the ChABC protein showed efficacy in glial scar degradation.^[^ ^29^ ^]^ However, the high doses coincided with side effects, including fatal encephalitis.^[^ ^29^ ^]^ Thus, ChABC protein is a promising enzyme, but its therapeutic potential is severely limited due to its poor stability and challenging therapeutic delivery. Recent approaches using directed protein engineering to improve the thermal half‐life of ChABC^[^ ^30^ ^]^ or using affinity‐based biomaterials to provide sustained protein delivery^[^ ^31^ ^]^ have also shown substantial improvements in ChABC treatment efficacy in SCI models. While these approaches represent significant advances in enzyme delivery within the central nervous system (CNS), they require engineering specifically for the ChABC protein. These kinds of approaches, therefore, would require redevelopment for other potential proteins of interest for augmenting ChABC delivery for spinal cord regeneration.

Gene therapy is an alternative strategy to deliver proteins with intrinsically poor stability (e.g., ChABC), as local protein production can allow for extended biological activity without required protein engineering or specialized delivery systems. In addition, Muir et al. previously identified a set of point mutations (mtChABC), which allows for the production and secretion of active ChABC from eukaryotic cells.^[^ ^32^ ^]^ Previously, Zhao et al. utilized a lentivirus vector to produce this mtChABC in the cerebral cortex and injured spinal cord of rats and demonstrated the impressive potential efficiency of a ChABC gene delivery strategy.^[^ ^33^ ^]^ However, efficient gene delivery in the CNS achieved via commonly used viral delivery methods is potentially limited by the immunogenic and insertional mutagenesis concerns presented by common viral vectors.^[^ ^34^ ^]^ Recently, messenger RNA (mRNA) delivery has emerged as an attractive strategy for non‐virally producing proteins in vivo with higher activity than recombinant proteins and a more desirable safety profile than viral gene delivery.^[^ ^35^, ^36^, ^37^ ^]^ The recent rapid development of mRNA‐based vaccines for infectious diseases such as SARS‐CoV‐2 illustrates the immense potential utility of this approach.^[^ ^38^, ^39^, ^40^ ^]^ However, short half‐lives of the mRNA and the need for repeated dosing current limit mRNA delivery approaches.^[^ ^41^, ^42^ ^]^

We have developed materials‐based on a non‐viral, single‐dosing method for mRNA delivery, in which mineral‐coated microparticles (MCMs) locally deliver mRNA encoding for a therapeutic protein of interest and then sequester the locally produced therapeutic protein within a wound site to sustain its biological effect.^[^ ^43^ ^]^ The MCMs have a unique ability to sequester and stabilize labile proteins^[^ ^44^, ^45^ ^]^ and are designed to dissolve and release their contents over an extended timeframe.^[^ ^44^, ^45^, ^46^, ^47^, ^48^, ^49^ ^]^ Specifically, we have previously shown that these mineral substrates can capture and sustain exogenous proteins produced in situ via mRNA delivery and that locally expressed proteins displayed higher activity than recombinant proteins.^[^ ^43^ ^]^ Additionally, we have utilized calcium‐based materials to previously deliver therapeutic proteins within the injured spinal cord.^[^ ^48^, ^49^ ^]^ As a result, we hypothesized that MCMs could deliver mtChABC‐encoding mRNA, leading to locally prolonged ChABC activity and, in turn, effective CSPG degradation in vivo.

We used in vitro biochemical assays to assess the activity of a mutant ChABC protein produced by eukaryotic cells, an in vitro astrogliosis model to determine CSPG reduction potential from mtChABC mRNA in primary glial cells, and a rat SCI model combined with the Basso, Beattie, and Bresnahan (BBB) Locomotion Scoring^[^ ^50^ ^]^ system to test our hypothesis. We also assessed the in vivo outcomes using immunohistochemistry of CSGP degradation in the glial scar and the presence of new axonal sprouting. Our results showed that MCM‐mediated mRNA delivery was an efficient non‐viral gene delivery method in primary neural cells in vitro and enabled localized transgene expression in the glial scar in vivo. We further demonstrated that MCM‐mediated delivery of mtChABC‐encoding mRNA resulted in decreased CSPG presence in an in vitro model of astrogliosis, as well as improved functional recovery in an established rat SCI model.

## 2 Results

### 2.1 MCMs and mRNA Result in Improved Transfection Efficiency and Transgene Expression in Primary Rat Glial Cells

MCMs effectively delivered lipoplexes of enhanced green fluorescent protein (EGFP)‐encoding plasmid DNA (pDNA) and mRNA to primary glial cells isolated from rat spinal cord (Figure 1B–E). Astrocytes and oligodendrocytes were the predominant cell populations in these transfected primary glial cells as shown by GFAP (astrocyte), nuclear Olig2 (Oligodendrocyte), Iba1 (microglia), and Tuj1 (neuron) immunocytochemistry (Figure 1C and Figure S1, Supporting Information). Both the use of MCMs and the choice of mRNA over pDNA increased the effectiveness of gene delivery, measured by the number of EGFP+ cells (Figure 1D,E). mRNA with chemically modified ribonucleases that reduce innate immunogenicity^[^ ^43^, ^51^ ^]^(CM‐mRNA) delivery resulted in the greatest number of EGFP+ followed by wild‐type (WT‐mRNA) and then little to no transfection using pDNA in equivalent numbers of cultured glial cells. Specifically, CM‐mRNA increased in EGFP+ glial cells 1.6‐fold relative to WT‐mRNA (Figure 1E). The additional use of MCMs for mRNA delivery increased the number of EGFP+ glial cells by 1.5‐fold for WT‐mRNA and by 1.7‐fold for CM‐mRNA (Figure 1E). At the optimal condition of 375 ng nucleic acid per well, the MCM‐mediated CM‐mRNA transfection resulted in a 200‐fold increase in the number of EGFP^+^ primary glial cells 24 h after transfection relative to pDNA without MCMs (Figure 1E).

### 2.2 Mutant Chondroitinase ABC Was Secreted, Enzymatically Active, and Reduced the Observed Chondroitin Sulfate in an In Vitro Model of Astrogliosis

**Figure 2:**
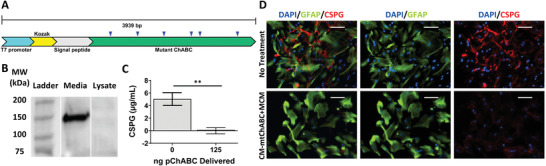
mtChABC overexpressed in eukaryotic cells is secreted and active for degrading chondroitin sulfate proteoglycan. A) Schematic representing template for mtChABC with T7 promoter for RNA synthesis, Kozak sequence for translation, mouse MMP‐9 signal peptide for secretion and point mutations listed in the methods section to prevent Golgi glycosylation and inactivation indicated with blue arrows. B) Western blot for ChABC from media supernatant and cell lysate from HEK293s transfected with the pDNA template from 6.2A driven by a PGK promoter. C) DMMB assay quantifying CSPG levels after CSPG digestion using cell supernatant from HEK293s transfected with the pDNA template from 6.2A driven by a PGK promoter. Mean + SD *N* = 3 **p‐value < 0.01 by Student's *t*‐test. D) Representative epifluorescence micrograph of untreated and CM‐mtChABC‐treated astrocytes in the astrogliosis model show reduced CSPG deposition with nuclei in blue, GFAP in green and CSPG in red. Scale bar = 50 µm

Delivery of mRNA encoding for a mtChABC CM‐mRNA (CM‐mtChABC) resulted in secretion of an active ChABC protein in eukaryotic cells (**Figure** **2**). ChABC is an enzyme derived from *Proteus vulgaris*,^[^ ^20^ ^]^ and as such, is not designed for expression in eukaryotic cells. A previous study identified multiple sites in the native protein that were subject to aberrant glycosylation in the Golgi of eukaryotic cells and inactivation, as well as a specific set of point mutations that allowed for the expression of a highly active form of the protein.^[^ ^52^ ^]^ An mRNA expression plasmid containing these point mutations, a T7 promoter, a Kozak sequence, and a signal peptide sequence from mouse matrix metalloproteinase 9 for secretion produced CM‐mtChaABC (Figure 2A and Figure S3, Supporting Information). Transfection and protein production from the expression plasmid in eukaryotic cells demonstrated mtChABC was both secreted and active (Figure 2A–C). Specifically, a western blot for ChABC recognized protein in the culture media but not in the cell lysate at the appropriate 120 kDa protein size (Figure 2B and Figure S3, Supporting Information), and degradation of 5 µg of CSPG A supplemented into the culture media of transfected cells demonstrated the protein was enzymatically active (Figure 2C). CM‐mtChABC transfection abrogated CSPG staining in an in vitro model of astrogliosis using transforming growth factor *β*1 (TGF*β*1)‐conditioned rat cortical astrocytes as shown in the reduction of red‐labeled CSPGs outside of the GFAP demarcated astrocyte cell bodies. (Figure 2D), demonstrating the feasibility of our mRNA approach to locally express active mtChABC from primary glial cells that was capable of degrading glial cell‐produced CSPGs.

### 2.3 MCMs Allowed for the Delivery of Biomolecules to the Spinal Cord via Direct Injection

**Figure 3:**
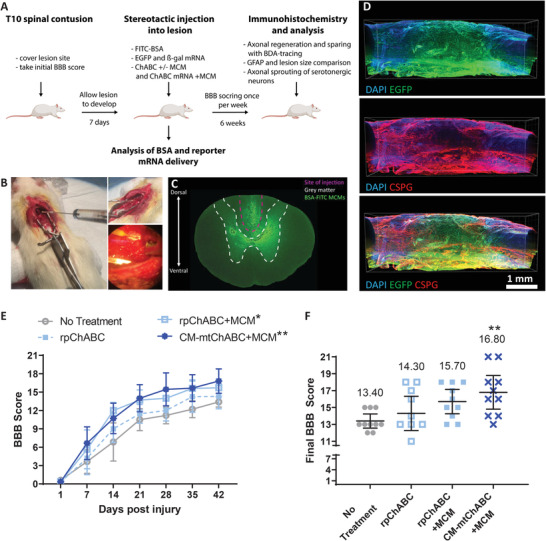
Stereotactic direct injection of settled MCMs allows for localized delivery of biomolecules in the spinal cord and improved motor function with CM‐mtChABC delivery. A) Schematic of study design. B) Stereotactic set up for direct injections into the spinal cord of an anesthetized rat having undergone a T10 laminectomy. Operating scope image showing injection (top) and settling of FITC‐BSA‐laden MCMs (bot) in glass needle. C) Transverse cryosection of injection site showing localized delivery and retention of FITC‐BSA at the injection site and surrounding grey matter. D) Confocal microscopy of whole‐mount immunohistochemistry of transfected SCI lesion showing volumes of chondroitin sulfate proteoglycan positive lesion and EGFP expression. E) BBB scoring of rats with SCI versus time with rats scored once per week. Mean + 95% CI N = 10 **, * *p*‐value < 0.01, 0.05, respectively, by two‐way ANOVA with Dunnett's post hoc analysis relative to no treatment control. F) Final BBB scores for each treatment group shows the greatest improvement for delivery of CM‐mtChABC via MCMs (CM‐mtChABC+MCM). rpChABC, with and without MCMs (rpChABC and rpChABC+MCM), did not show a significant improvement relative to the control. Mean + 95% CI N = 10 **p‐value < 0.01 by two‐way ANOVA with Dunnett's post hoc analysis relative to no treatment control.

Using a stereotactic, we developed a model system to locally deliver a variety of biomolecules to a contused spinal cord to facilitate our study design (**Figure** **3**A). Stereotactic microinjection of MCMs afforded localized delivery of biomolecule cargo in the spinal cord (Figure 3B). Specifically, MCMs bound fluorescein‐conjugated bovine serum albumin (FITC‐BSA) out of an aqueous solution, and subsequent stereotactic injection of the MCMs allowed for localized delivery of the cargo. By drawing the FITC‐BSA‐laden MCM solution into a pulled glass syringe and then allowing them to settle to the tip of the syringe before injection, the MCM‐delivery approach provided a facile method to deliver a larger amount of cargo with smaller injection volumes (Figure 3B). Fluorescence imaging of fixed tissue sections revealed that FITC‐BSA was abundant within the injection site but not in the ventral white matter (Figure 3C) or regions distal and caudal to the injection site, indicating localized biomolecule delivery.

Stereotactic injection of MCMs containing either ß‐galactosidase‐encoding CM‐mRNA (CM‐mßGal) lipoplexes or EGFP CM‐mRNA (CM‐EGFP) lipoplexes one‐week post contusion at the T10 vertebrae demonstrated localized transgene overexpression within an injured spinal cord (Figure 3D and Figure S2, Supporting Information). Upon stereotactic CM‐EGFP delivery via MCMs, green fluorescence localized to the injection site coupled with the abundant presence of CSPG indicated localized EGFP production within a glial scar. (Figure 3D). Similarly, upon CM‐mßGal delivery via MCMs, staining for the degradation product D‐galactose demonstrated localized production of an active ß‐Galactosidase enzyme at the injury site (Figure S4, Supporting Information). Additionally, the lack of positive ß‐galactosidase activity in any control sections or in any sections caudal and rostral to the injury/injection site provided additional evidence of the highly localized transgene production (Figure S4, Supporting Information).

### 2.4 MCM‐Mediated Delivery of CM‐mtChABC Resulted in CSPG Digestion, Sprouting of Serotonergic Axons, and Improved Hind Limb Motor Function Post Spinal Cord Injury

A 10‐gram weight was dropped on the rat spinal cord at the T10 level from a height of 12.5 mm, creating a mild/moderate SCI, which resulted in hindlimb paraplegia providing a testbed to evaluate different ChABC treatment modalities’ efficacy in functional motor recovery (Figure 3A).^[^ ^53^ ^]^ Local delivery of the treatments one week post‐injury via stereotactic microinjection additionally allowed for evaluation of glial scar degradation and any impact on axons. A saline control (No Treatment), MCM alone vehicle control (MCM Only), and recombinant ChABC protein (rpChABC) delivered via MCMs provided comparison groups for our CM‐mtChABC+MCM delivery approach (Figure 3A). Use of the Basso, Beattie, and Bresnahan (BBB) Locomotion Scoring^[^ ^50^ ^]^ over six weeks provided a standardized scoring basis to compare hindlimb functional improvements between treatment groups. Injection of biotinylated dextran amine (BDA) in the red nucleus and reticular formation six weeks after injury and followed by three additional weeks for axon transport of the dextran^[^ ^54^ ^]^ allowed for assessing axons that remained intact after injury. Immunohistochemistry for intact CSPG and its degradation productions provided an output for assessing the treatment groups’ effect on glycosaminoglycan digestion and immunohistochemistry for 5‐hydroxytryptamine (5‐HT) transporter labeling of axonal projections allowed for the assessment of differences in axonal sprouting and growth serotonergic neurons (Figure 3A).

**Figure 4:**
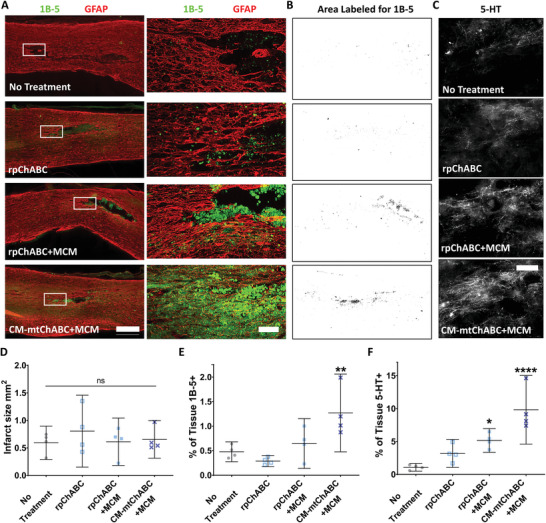
MCM‐mediated CM‐mtChABC delivery promoted glial scar degradation in rats with SCI and sprouting of serotonergic neurons. A) Representative images showing digested CSPGs labeled with 1B‐5 (green), counterstained for astrocytes with GFAP (red), and B) representative automated analysis threshold images of 1B‐5 positive area. C) Representative images showing 5‐HT positive serotonergic neurons sprouting at the rostral injury edge. D) Analysis of lesion infarct size measured by area outlined by GFAP analysis. Mean + 95% CI N = 4 ^ns^ *p*‐value > 0.05 by one‐way ANOVA with Dunnett's post hoc analysis relative to no treatment control. E) Immunohistochemical analysis of degraded chondroitin sulfate products (1B‐5). Mean + 95% CI N = 4 **p‐value < 0.01 by one‐way ANOVA with Dunnett's post hoc analysis relative to no treatment control. F) Immunohistochemical analysis of 5‐HT positive serotonergic axon sprouting. Mean + 95% CI N = 4 *,****p‐value < 0.05, 0.0001 by one‐way ANOVA with Dunnett's post hoc analysis relative to no treatment control. Scale bars: (A) = 500 µm, (B,C) = 50 µm.

Rats treated with CM‐mtChABC+MCM resulted in the greatest restoration of motor function (Figure 3E,F) relative to the three control groups, as measured by BBB score. Of the treatments explored, only CM‐mtChABC+MCM and rpChABC+MCM treatments resulted in a faster rate of BBB score improvement relative to no treatment (Figure 3E). However, the rpChABC+MCMs did not offer a significant improvement when compared to the MCM‐only treatment (Figure S5A, Supporting Information). At the conclusion of the animal monitoring period, the CM‐mtChABC+MCM treatment resulted in a 3.4 point improvement in the BBB score relative to the saline control and was the only significant improvement among the treatment groups (Figure 3F and Figure S5B, Supporting Information). Histologically, the infarct size at the end of the six‐week recovery period was consistent between all groups (**Figure** **4**D), and we observed no differences in the remaining intact axons caudal to the injury site, showing that the extent of tissue damage from the contusion was similar across all groups.(Figure S6, Supporting Information). For proteoglycan composition, we observed no differences in the amount of intact CS56‐stained CSPGs between (Figures S7 and S8, Supporting Information), but the CM‐mtChABC+MCM treatment resulted in significantly more 1B‐5 digested CSPGs within the injury site relative to no treatment (Figure 4A,B,E and Figure S9, Supporting Information). For neuronal outcomes, we observed 5‐HT positive axons rostral to the injury in all treatment groups (Figure 4C,E). However, only the rpChABC+MCMs and CM‐mtChABC+MCM treatments significantly increased additional axonal sprouting and growth. The rpChABC+MCM and CM‐mtChABC+MCM treatments resulted in 6.5‐ and 12.7‐fold increases, respectively, in sprouting serotonergic neurons relative to the no treatment control. (Figure 4C,E and Figure S10, Supporting Information). We observed no apparent differences in inflammation between the CM‐mtChABC+MCM treatment group and the no treatment control as indicated by similar levels of infiltrating amoeboid macrophages (Figure S11, Supporting Information).

## 3 Discussion

SCIs establish a chronic wound (Figure 1A) with no curative treatment strategies currently available.^[^ ^12^ ^]^ Several strategies have been explored to deliver anti‐inflammatory, neurotrophic and glial scar degrading proteins, but protein‐based therapeutics are often challenging to deliver due to poor and/or rapidly lost bioactivity.^[^ ^55^ ^]^ Non‐viral gene delivery is an alternative strategy to produce highly bioactive proteins locally and circumvent challenges associated with protein‐based therapeutics. However, gene delivery to the CNS has been notoriously difficult to accomplish.^[^ ^56^ ^]^ Previously, we demonstrated an effective non‐viral mRNA‐based, topical delivery strategy to upregulate a pro‐healing growth factor in an in vivo dermal chronic wound environment.^[^ ^43^ ^]^ Here, we adapted this approach for the CNS using direct glial scar injection of MCMs loaded with mRNA lipoplexes encoding for exogenous glial scar‐modifying protein, ChABC. The lipoplex loading and stereotactic injection‐based approach afforded transition of the topical‐based mRNA gene therapy approach to a localized internal environment present with additional challenges such as the high rates of dynamic cerebrospinal fluid flow. In vitro, MCM‐mediated transfection via mRNA delivery resulted in superior transfection in primary cells in terms of the overall number of EGFP+ cells when compared to pDNA (Figure 1D,E), consistent with our previous findings. However, here, MCM‐mediated delivery further improved mRNA delivery, ultimately showing up to a 200‐fold increase in the number of transfected cells relative to an equivalent dose of pDNA (Figure 1E). Lastly, this materials‐based approach represents, to our knowledge, the first effective non‐viral and local delivery of mRNA to a spinal cord lesion in the CNS.

Direct injection of fluids into the CNS often results in shear damage due to the compliant nature of the tissue.^[^ ^57^ ^]^ In addition, the delivered cargo is often quickly lost due to transport via cerebral spinal fluid.^[^ ^57^ ^]^ These properties of the CNS limit both the total volume injected and the injection rate, which limits the amount of cargo that can be delivered without significant damage to the tissue surrounding the injection site.^[^ ^57^ ^]^ As a result, therapeutic molecules must be highly concentrated for injection into the CNS in order to minimize the injection volume. However, lipoplex formation requires low concentrations of nucleic acid and a cationic lipid (≈10 ng µL^−1^).^[^ ^58^ ^]^ This requirement is a particular challenge for non‐viral gene delivery in vivo, as effective doses on the order of micrograms are required to elicit a therapeutic effect.^[^ ^59^ ^]^ Here we demonstrated a technique for delivering high concentrations of therapeutic molecules via direct injection, using the MCMs to concentrate the molecules (Figure 3). Specifically, MCMs are capable of binding biological molecules out of aqueous solutions via electrostatic interactions,^[^ ^44^, ^45^, ^46^, ^49^, ^60^, ^61^, ^62^ ^]^ and the density of the MCMs allows them to rapidly settle to the bottom of a syringe. Based on our previous studies^[^ ^43^, ^62^ ^]^ demonstrating a greater than 80% lipoplex binding efficiency of MCMs, concentrating 750 ng of mRNA in 25 µL MCM‐lipoplexes into the fixed 5 µL injection increased the amount of lipoplexes deliverable by up to 4‐fold. This approach using MCMs to bind and concentrate a therapeutic molecule in a fixed volume injection (Figure 3B,C) represents a simple yet efficient method to locally deliver relatively high doses of molecules to the CNS via direct injection.

Spinal cord secondary injury and glial scar formation involve a complex cascade of local inflammation, neurotoxicity, astrocyte proliferation, and extracellular matrix remodeling.^[^ ^6^ ^]^ Previous studies have established that the glial scar in the rat spinal cord contusion model used in our current study becomes substantial within 6–8 days post‐injury.^[^ ^63^ ^]^ Thus, the rat spinal contusion model was appropriate to address our hypothesis that MCM‐mediated delivery of CM‐mtChABC could degrade an established glial scar. More specifically, we sought to determine whether we could create the injury, then return one week later and deliver CM‐mtChABC via local, stereotactic injection of concentrated MCMs within a glial scar. Through direct injection of MCMs delivering CM‐mßGal or CM‐mEGFP our results established that injection of mRNA‐laden MCMs caused localized transfection within the injury site of the contused spinal cord (Figure 3D and Figure S2, Supporting Information). This finding was significant, as previous studies have shown that non‐localized delivery of ChABC in the spinal cord can result in severe side effects, including death.^[^ ^29^ ^]^

We used multiple cell‐based and biochemical‐based assays to directly observe the activity of the mtChABC produced by non‐viral transfection. First, we expressed mtChABC via pDNA in primary human cells (HEK293s) and showed that the protein was reactive to antibodies raised against wild‐type ChABC, was secreted, and was active in degrading chondroitin sulfate (Figure 2B,C). In addition, we generated CM‐mRNA from the mtChABC expression plasmid (Figure S2, Supporting Information). Transfection of CM‐mtChABC via MCMs decreased CSPG observed (Figure 2D) in a TGF‐*β*‐mediated in vitro model of astrogliosis that mimics what occurs in a spinal cord following trauma.^[^ ^8^ ^]^ While this approach demonstrated that the MCM‐mediated CM‐mtChABC delivery to glial cells could reduce CSPG, future work might aim to quantify the amount of protein that can be produced from this approach as well as compare the bioactivity of the rpChABC versus the mtChABC produced locally by the cells after mRNA transfection. For example, local, in situ protein production via mRNA would be expected to produce a higher ratio of active to denatured protein than recombinant production.^[^ ^43^, ^64^ ^]^ Also, posttranslational modifications such as protein glycosylation absent in recombinant bacterial systems can influence ChABC activity.^[^ ^32^ ^]^ Additionally, we have previously demonstrated that mineral coatings can deliver neural growth factors and cytokines within the neural tissue.^[^ ^48^, ^49^ ^]^ The MCM‐mediated mRNA delivery method in our current study might be explored in combination with these other neurotrophic factors in future studies to simultaneously reduce the glial scar and promote axonogenesis, similar to our recent studies exploring biomaterial delivery of mRNA for local production of growth factors in chronic wounds.^[^ ^43^ ^]^

In addition to validating that the MCM‐mediated mRNA delivery could result in effective in situ enzyme production within a glial scar and produce active ChABC from eukaryotic cells, the results here showed the same biomaterial delivery system delivering mRNA outperformed the ChABC protein manufactured via recombinant DNA technologies (“recombinant ChABC”). Specifically, recombinant ChABC did not yield the same improvement in functional recovery (Figure 3E,F) and resulted in less CSPG degradation (Figure 4A,B,E) and sprouting of serotonergic neurons (Figure 4C,F) when compared to mRNA encoding for ChABC. This outcome illustrates the potential advantages of delivering nucleic acids relative to recombinant proteins as a therapeutic strategy in the CNS. Moreover, the nucleic acid‐based nature of the approach described here could be used to deliver a different protein by simply changing the mRNA transcript. In contrast, recombinant protein engineering or affinity‐based delivery systems would potentially require complete redevelopment to deliver a new protein.

In another important distinction, the approach described here did not employ viral vectors like previously published gene delivery strategies to locally produce ChABC in the CNS. An effective mRNA‐based approach provides a benefit over viral‐ and DNA‐based strategies by considerably lowering safety concerns of immunogenic responses and insertional mutagenesis. From a therapeutic development perspective, the preparation of cationic lipoplexes and electrostatic adsorption onto our biomaterials represents a simpler preparation method than viral vectors. In addition, simply changing the mRNA transcript to deliver an alternative protein is considerably easier than designing and producing a new viral vector. Thus, the non‐viral, biomaterial‐mediated mRNA delivery strategy in this study represents an effective and generalizable approach to produce therapeutic proteins within the CNS with distinct advantages when compared to previous recombinant protein‐ or virus‐based strategies. While many of these biologic effects could not be achieved via recombinant protein biologics (i.e., intracellular proteins such as transcription factors), future studies should include controlled comparisons of dosage, duration and biological activity between recombinant proteins and those produced in situ via non‐viral mRNA delivery.

We evaluated functional recovery after SCI in response to the different treatment methods via blinded behavioral monitoring and using the established BBB^[^ ^50^ ^]^ locomotor scale in rats. This analysis examines the overall use of the hindlimbs, articulation of the ankle, knee and hip, as well as coordination of the hindlimb with the opposing forelimb during locomotion.^[^ ^50^ ^]^ Here, the rats received a moderate contusion with a 10‐gram weight drop from a 12.5 mm height. Although rats with this moderate injury progress from little to no hindlimb movements to consistent stepping, they are unable to coordinate the forelimbs and hindlimbs consistently.^[^ ^53^ ^]^ In this study, the recovery of the untreated control rats plateaued at the high end of the intermediated stage of recovery, while the MCM‐mediated delivery of CM‐mtChABC treatment group did significantly better and plateaued in the middle of the late stage of recovery (Figure 3E,F and Movies S1 and S2, Supporting Information). While some of the rats in the CM‐mtChABC treatment group recovered to a perfect BBB score, it is important to note that the BBB scoring method is based on the rat walking in an open field at its own speed and does not take into account the skills needed for other activities such as running or climbing. Thus, it is likely that even if a rat recovers to a BBB score of 21, it would still have noticeable coordination impairments if assessed on tests such as beam walking or the ladder rung test. The improvement in functional recovery observed here is consistent with previous work showing that digesting CSPGs after SCI with rpChABC increases axonal sprouting and growth, although this effect required repeated delivery over the course of 10 days.^[^ ^65^, ^66^ ^]^ This sprouting has been shown to improve functional recovery, including manual dexterity and forelimb stepping after cervical injuries,^[^ ^19^, ^66^, ^67^ ^]^ bladder function after thoracic injuries,^[^ ^68^ ^]^ and hindlimb locomotion after thoracic injuries.^[^ ^27^, ^68^, ^69^, ^70^, ^71^ ^]^

The extent to which functional recovery occurs after ChABC treatment in previous studies varies significantly. This variation is likely due to treatment time after injury, ChABC dosage, and mode of delivery. Similar to Lee et al.,^[^ ^27^ ^]^ we did not observe functional recovery from a single treatment of rpChABC with or without a sustained release from the MCMs. However, we did observe a trend in increased function in the rpChABC+MCM and a significant increase in serotonergic axonal sprouting, suggesting a subtle effect of sustained release of ChABC in a single‐dose regimen. In contrast, the combined observed significant improvement in functional recovery via the MCM‐mediated delivery of CM‐mtChABC, suggesting, similar to our previous findings,^[^ ^43^ ^]^ a higher activity of locally produced proteins in situ as well as potential sequestration and sustained activity of the overexpressed protein in the injury site. Taken together, these data suggest that the influence of CM‐mtChABC delivery on the glial scar led to the improvement in functional recovery, even in the absence of any direct therapeutic stimulator of axonal growth. Future studies may further improve functional recovery by combining ChABC activity with therapies to stimulate axonal growth, such as neural growth factors or cell therapies,^[^ ^10^, ^27^, ^48^, ^71^, ^72^ ^]^ as well as including additional functional testing such as the ladder rung^[^ ^73^ ^]^ test for more granular analysis of hindlimb function. Lastly, the improvements observed here were achieved with a single‐dose intervention. Repeated deliveries are an additional future direction worth exploring.

Histological analysis revealed that, although there was no difference in the amount of intact CSPGs between the groups (Figures S7 and S8, Supporting Information), there were significantly more digested CSPGs in the rats that received MCM‐mediated treatment with CM‐mtChABC (Figure 4A,B,D). This lack of difference in the amount of intact CSPGs can be explained by previous observations that CSPGs are known to be upregulated for weeks after injury,^[^ ^74^ ^]^ and it is likely that new CSPGs were being continuously produced after application of the different treatments. As expected, based on our axon tracing analysis, there were no significant differences in terms of axon‐sparing through the injury site among treatment groups. GFAP labeling also revealed no difference in infarct size (Figure 4D), suggesting that the contusions were similar among rats and that the one‐week scar formation period between injury and treatment caused permanent axonal damage. When assessing serotonergic axons, we observed significantly more 5‐HT positive axons in the rats that received MCM‐mediated treatment with CM‐mtChABC (Figure 4C,E). This finding of increased serotonergic axon sprouting in response to CSPG degradation is consistent with the previous literature.^[^ ^23^, ^67^, ^75^, ^76^ ^]^ Thus, the observed digestion of the CSPGs in the glial scar is the most likely reason for the improvement in hind limb function in the CM‐mtChABC treated animals via the increase in neuronal sprouting near the injury site. This proposed mechanism is in line with previous studies, which have shown that digesting CSPGs increases axonal sprouting and growth, and leads to increased functional recovery after complete spinal cord transection,^[^ ^77^, ^78^ ^]^ incomplete sharp cut injuries^[^ ^27^, ^65^, ^66^, ^67^, ^79^, ^80^ ^]^ and spinal cord contusion injuries.^[^ ^68^, ^69^, ^70^, ^71^ ^]^ Future directions should examine additional histological mechanisms such as CSPG‐degradation mediated axonal sprout myelination^[^ ^81^ ^]^ and the nature of new synaptic formation in response to the mRNA approach described here.

## 4 Conclusion

Previous studies have demonstrated the advantages of gene delivery over protein delivery,^[^ ^82^ ^]^ and non‐viral gene delivery to neural tissue has been notoriously challenging.^[^ ^56^ ^]^ The method developed here provides an effective method for delivering therapeutic mRNA and overexpressing an exogenous protein in the spinal cord. The characteristics of the approach allow for the localized expression of mRNA gene products with high levels of biological activity. Delivery of mRNA encoding for a eukaryotic mutant ChABC—a known effector of glial scar degradation—led to localized CSPG degradation and improved functional recovery in an established SCI. Future work might explore the addition of mRNA encoding for chemokines and neurotrophic factors in addition to ChABC, as degradation of the glial scar alone is not likely to be sufficient to promote complete neuronal regeneration in larger animal models.^[^ ^63^, ^83^ ^]^ In addition, the method developed in this study could be explored in other regions of the CNS, where gene delivery has proven challenging.^[^ ^84^ ^]^

## 5 Experimental Section

### Fabrication of MCMs

Hydroxyapatite powder (Plasma Biotal Limited) was used as a microparticle core material. The powder was suspended at concentrations of 1 mg mL^−1^ in modified simulated body fluid (mSBF) containing concentrations of: 141 mm NaCl, 4 mm KCl, 0.5 mm MgSO_4_∙7H_2_O, 1 mm MgCl_2_∙6H_2_O, 4.2 mm NaHCO_3_, 20 mm HEPES, 5 CaCl_2_∙2H_2_O, 2 mm KH_2_PO_4_, and 1 mm NaF. The suspension was rotated at 37 °C for 24 h, at which point the microparticles were centrifuged at 2000 g for 2 min, and the supernatant decanted and replaced with freshly made mSBF. We repeated this process daily for 5 days, at which point the MCMs were washed three times with 50 mL deionized water, filtered through a 40 µm pore cell strainer, suspended in 15 mL distilled water, frozen in liquid nitrogen and lyophilized for 48 h. The lyophilized MCMs were then analyzed for nanotopography and calcium release as previously described.^[^ ^46^, ^85^ ^]^

### Glial Cell Isolation and Culture

Ten‐week‐old ≈225 g female Sprague Dawley rats (Charles River) were euthanized with a lethal dose of isoflurane and were perfused transcardially with 0.9% saline until all blood was removed. The animal was decapitated in the cervical spine using a rodent guillotine, and the hindquarters were removed at the lumbar spine in the same manner. A sterile 16 gauge needle was fitted onto a sterile syringe filled with sterile saline and placed at the exposed rostral base of the spine. Pressure was applied gradually on the syringe plunger to hydrodynamically eject the spinal cord from the exposed region of the caudal spine. The spinal cord was then collected and rinsed in sterile ice‐cold Hank's Balanced Salt Solution (HBSS) with 100 U Penicillin/Streptomycin 3× by immersion. The rinsed spinal cord was then placed in a sterile petri dish and mechanically digested for 5 min with surgical scissors until a putty‐like consistency was achieved. The mechanically digested spinal cords were added to 5 mL of 0.25%Trypsin /EDTA (Corning) and manually triturated with a 5 mL plastic pipetted 30 times, taking care not to introduce bubbles.

The triturated spinal cords were incubated at 37 °C on a plate shaker for 30 min, followed by an additional trituration of 30×. The digest was quenched with 20 mL Dulbecco's Modified Eagle's Medium (DMEM) + 10% FBS and centrifuged at 200 x *g* for minutes. The supernatant was aspirated and replaced with 5 mL DMEM + 10% FBS and triturated 30× more. 20 mL DMEM + 10% FBS was added and centrifuged a second time. 5 mL astrocyte growth media (DMEM High Glucose + GlutaMAX + N2 supplement + 10% FBS + 100 U Penicillin/Streptomycin (All Gibco),+10 ng mL^−1^ rat epidermal growth factor (Gold Bio) and triturated 30× more. Corning poly‐D‐lysine Biocoat culture dishes were used for all astrocyte cultures. They were additionally coated with mouse‐derived laminin (Sigma Aldrich) at 2 µg mL^−1^ for 1 h at room temperature prior to use. 25 mL growth media was added to the spinal cord cell isolate, and 10 mL was seeded 3× in laminin‐coated T‐75 PDL‐Laminin Biocoat flasks. The cells were cultured for 5 days and then placed on a shaker at 37 °C for 3 h to dislodge loosely attached cells. The media was aspirated and aggressively washed with fresh culture media via pipetting and manually tapping of the flask. The washing media was aspirated and replaced with growth media. These isolated cells were deemed glial cells and subcultured in outgrowth media. Cells were passaged when 80% confluence was reached via removal of growth media, wash 1× in 1× PBS, and then incubated for 5 min 0.25% Trypsin/EDTA. Rat cortical astrocytes were commercially purchased (Lonza) and subcultured in the same manner except were passaged in 0.05% Trypsin/EDTA (Corning).

### mRNA Synthesis

mtChABC ORF plasmid was designed in Benchling Molecular Biology Suite with the following mutations: Asn‐751 (N‐Q), Ser‐517 (S‐A), Asn‐345 (N‐Q), Asn‐282, (N‐K), Asn‐675 (N‐Q) as described in Muir et al.^[^ ^52^ ^]^ and ordered as‐synthesized plasmid (Genscript). The mtChABC ORF was cloned into pPGK‐Puro (Addgene #11 349) via Gibson Assembly (GA Master Mix‐New England Biolabs) with the following primers: mtChABC FWD‐TCGAGCAGCTGAAGCTTACCGCCGCCATGGAGGCAAGAGT, mtChABC REV‐CCCGGGGATCTGATATCATCTCACGGCAGAGGGGACAGCT, pPGK‐Puro FWD‐AGCTGTCCCCTCTGCCGTGAGATGATATCAGATCCCCGGG and pPGK‐Puro REV‐ACTCTTGCCTCCATGGCGGCGGTAAGCTTCAGCTGCTCGA (IDT) to generate pPGK‐mtChABC with mtChABC under the PGK promoter for eukaryotic expression from plasmid transfection. pPGK‐mtChABC was cloned in DH5alpha chemically competent *Escherichia coli*, selected against 50 µg mL^−1^ Ampicillin‐agar plates, amplified in 50 µg mL^−1^ Ampicillin‐LB broth and purified via silica columns (GeneJet Miniprep‐Thermo Fisher). The size of the construct and digest were analyzed on 1% agarose gel. For mRNA synthesis, the following primers were used to amplify mtChABC form pPGK‐mtChABC and add a T7 promoter: T7 mtChABC FWD‐ACCTGCAGCCAATTAATACGACTCACTATAGGGGCTTACCGCCGCCATGGAGG, and T7 mtChABC REV‐TCACGGCAGAGGGGACAGCT, and followed with 1 h treatment with DpnI (New England Biolabs) to remove plasmid template. The T7 mtChABC PCR template for mRNA synthesis was analyzed on a 1 wt% agarose gel in TAE buffer at 8 V cm^−1^ and then used directly with T7 HiScribe + ARCA mRNA synthesis kit with poly‐A tailing (New England Biolabs) supplemented with 5‐methyl cytosine and Psuedouridine (Tri‐Link Biotechnologies) according to the synthesis kit instructions. mRNA was purified via silica column (Zymogen RNA Cleanup 100) and analyzed on an agarose gel as follows: mRNA was first denatured via incubation at 70 °C with NorthernMax Glyoxal‐DMSO (Thermo Fisher) for 5 min and immediately placed on ice. Denatured mRNA was run in 1.5 wt% agarose gels in MOPS running buffer (Fisher Scientific) at 5 V cm^−1^ and 4 °C with a circulating pump constantly circulating buffer between chambers. These steps were critical to resolve mRNA gels. Wild‐type (WT) and chemically modified (CM) mRNA encoding for EGFP were purchased commercially (Tri‐Link Biotechnologies).

### In Vitro Transfection of Glial Cells

Rat cortical astrocytes (Lonza) and isolated primary RSAs were seeded in 96‐well cyclic‐olefin high content PDL (Corning Biocoat) plus laminin (as described above in the isolation of primary glial cells) plates at 15 000 cells/cm^2^ 36–48 h prior to transfection in growth media. Wild‐type (WT) and chemically modified (CM) mRNA encoding for EGFP were complexed with Lipofectamine Messenger Max (Invitrogen) at 30 µg mL^−1^ in OPTI‐MEM (Gibco) with a ratio of 3 µL of Lipofectamine Messenger Max per µg of mRNA. pDNA encoding for EGFP‐N1 (Clontech) was complexed with Lipofectamine 2000 at 30 µg mL^−1^. The mRNA and DNA complex solutions were incubated for 20 min at room temperature to allow for complexes to form. Cells were transfected with 100 ng of mRNA complexes (by mass mRNA) unless otherwise specified. For MCM transfections, mRNA or pDNA lipoplexes were added to MCMs at a ratio of 1 µg of nucleic acid to 13.3 µg MCM and incubated under constant rotation at room temperature for 30 min. After 30 min, the MCM+ and MCM‐lipoplexes were added directly to the cell culture media. Five hours post‐transfection, the transfection media was removed and replaced with growth media to promote cell survival. Cells transfected with EGFP mRNA and pDNA were examined for green fluorescence using epifluorescence microscopy (Nikon Ti Eclipse with FITC filter cube) 12 and 24 h post‐transfection.

### Western Blot

HEK293s (AllCell) were transfected with pPGK mtChABC as described above (transfection of glial cells), except DMEM + 10% FBS and 100U Penicillin/Streptomycin was used in place of astrocyte growth medium. Thirty‐six hours post‐transfection, cell supernatant was collected and stored and placed on ice as “Media”, and the cells washed with PBS. Cells were lysed ice‐cold RIPA buffer containing 1× Halt Protease/Phosphatase Inhibitor Cocktail and stored on ice as “Cell Lysate.” Media and Cell Lysate protein fractions were concentrated using Amicon Ultra 4 mL Centrifugal filters and protein concentration determined via UV absorption at 280 nm and then stored at −20 °C. For the western blot, equal amounts of total protein per fraction were combined with Laemmli buffer, denatured for 5 min at 100 °C, loaded in 10% polyacrylamide gels, and separated by SDS‐PAGE. Proteins were transferred to PVDF membranes and incubated in blocking buffer (5% nonfat dry milk in TBST) for 1 h at RT. Membranes were incubated in primary antibodies in blocking buffer overnight at 4 °C, washed with TBST, and incubated in horseradish peroxidase (HRP)‐conjugated goat anti‐mouse IgG secondary antibody in blocking buffer (Abcam, 1:10 000) for 1 h at RT. Membranes were washed with TBST and incubated with ECL Western Blotting substrate (Pierce) for 6 min. Chemiluminescence was detected using a LAS4000 Mini imager. The primary antibody and dilution used was mouse anti‐chondroitinase ABC (Novus Bio NBP1‐96142‐1:300).

### DMMB Assay

95 µL of cell supernatant was collected from HEK293 transfection of pPGK‐mtChABC (as described in western blot methods) and placed into a 96‐well multiwell plate. 5 µg of Chondroitin Sulfate A sodium salt (Sigma Aldrich) in 5 µL PBS was added to the supernatant from transfected and untransfected cells and incubated for 1 h. Dimethylmethylene blue (DMMB) (Sigma Aldrich) was dissolved as following in 1 L DI water: 16 mg DMMB, 3.04 glycine, 1.6 g NaCl, 95 mL 0.1 m Glacial acetic acid, and pH adjusted to 3.0 with 2 m HCl. 50 µL of the supernatants were added to 150 µL of the DMMB working solution and shaken at a speed of 600 for 30 s. The plate was read immediately for absorbance for each well at 525 nm. Means of 3 replicates are reported with standard deviation. A Student's t‐test was performed between transfection and no transfection supernatant absorbances with significance determined as *p* < 0.05.

### In Vitro Reactive Astrogliosis

Rat cortical astrocytes and isolated primary RSAs were seeded at 15 000 cells/cm^2^ in 96‐well cyclic‐olefin high content PDL (Corning Biocoat) plus laminin. Cells were grown to confluence (24–48 h), and growth media was replaced with TGFß1 induction media (growth media ‐ FBS + 10 ng mL^−1^ TGFß1) for 5 days with induction media replaced with fresh media on the third day. On day 6, 100 ng CM‐mtChABC lipoplexes were in 25 µL OPTI‐MEM as well as 25 µL blank OPTI‐MEM as a no treatment control. 48 h post‐treatment, cells were then in neutral buffered formalin, washed 3× in 1× PBS, and then stored in 1× PBS at 4 °C for immunocytochemistry.

### T10 Spinal Cord Contusion

The University of Wisconsin—Madison animal care and use committee approved all procedures, which followed the NIH Guide for animal care. Ten‐week‐old ≈225 g female Sprague Dawley rats (Charles River) were anesthetized with a combination of xylazine 5–10 mg kg^−1^ and ketamine 50–100 mg kg^−1^, injected intraperitoneal (IP). Ten rats were used per group. The T10 vertebral spinous process and laminae were surgically resected, exposing the dorsal dura covering the spinal cord. Rats were fixed in place by clamping the spinous processes rostral and caudal to the laminectomy and then slightly suspended by these clamps to prevent movement of the spinal cord due to respiration. The rats were positioned below the weight drop machine (MASCIS Impactor Model II). The 10 g impactor rod was carefully lowered to align the rod with the exposed dura of the spinal cord, and then the rod was raised to a 12.5 mm height and released to contuse the spinal cord. After contusion, a small piece of non‐Latex neoprene sterile surgical glove (Gammex) was cut and placed over the exposed spinal cord before the muscle was closed with 4‐0 Vicryl sutures and the skin was closed using 4‐0 nylon sutures. The sterile glove was placed on the spinal cord to reduce scar tissue adhering to the dura mater; thus, making the injury site easier to locate and clean for the 7‐day post‐injury injections. For pain management, rats were given a subcutaneous injection of buprenorphine 0.05 mg kg^−1^. To prevent infections, rats were given Harlan Commercial Uniprim diet for 7 days. The resulting diet contains 275 ppm trimethoprim and 1365 ppm of the sulfonamide sulfadiazine. All rats were housed in twos per cage, and their bladders were expressed twice daily until the function was regained.

### Stereotactic Injection into Spinal Cord

CM‐mtChABC, CM‐mßGal, and CM‐mEGFP mRNA were prepared with MCMs as in 5.4. 100 µg of MCMs were added to either 7.5 µg of CM‐mRNA lipoplexes or 5 µg rpChABC or BSA‐FITC and incubated for 30 min at room temperate under constant rotation. No treatment featured a mock injection, and MCMs with no cargo was used as a vehicle control. Seven days post‐contusion, rats were anesthetized as described in 5.8, the injury site was reopened, and the piece of sterile glove was removed. The rats were fixed in place by clamping the spinous processes rostral and caudal to the laminectomy and then positioned under a 25 µL glass syringe (Hamilton) held in a stereotaxic (Stoelting). The rodent was suspended by these clamps to prevent movement of the spinal cord relative to the syringe during respiration. The syringe tip was pushed through caulk‐filling in the blunt end of pulled‐glass needles (≈100 µm diameter tip) and then backfilled with mineral oil to displace the air. For MCM conditions, 25 µL of the solutions was drawn up into the glass syringe, and MCMs were allowed to settle for 1 min prior to injection to concentrate the MCMs in the needle tip. The needle was lowered 1 mm through the dura into the epicenter of the injury, and the contents injected intramedullary at 1 µL per 30 s for a total of 5 µL. After the injection, the needle was removed, the animal released from the holding clamps, and the wounds sutured closed. The animals were monitored until they recovered from anesthesia.

### BBB Scoring

The BBB scoring method is a 21‐point scale previously described here.^[^ ^53^ ^]^ Briefly, 9–10 rats from each treatment group were placed in an open field and videotaped for 4 min. Another observer scored the hindlimb movements according to the parameters previously described for the BBB criteria.^[^ ^53^ ^]^ To ensure a proper spinal cord contusion, all rats were assessed 24 h after injury, and any rat with a BBB score of 3 or higher was considered an atypical injury and immediately removed from the study. To assess recovery, scores were taken for each animal every week post‐injury for 6 weeks. Handlers and scorers were blinded to the animal treatment group throughout the study.

### Axon Tracing

Six weeks after SCI, the anterograde axon tracer BDA (Thermo Fisher D1956) was injected bilaterally into the red nucleus and reticular formation of the brainstem in 4–5 animals per treatment group at a concentration of 10% BDA in sterile saline.^[^ ^54^ ^]^ The following stereotaxic coordinates were used for craniotomy and BDA injection, using the bregma as the zero point: red nucleus sites: anterior–posterior 5.8 mm, medial–lateral ± 1.2 mm, depth from the dural surface 7 mm; and reticular formation sites: anterior–posterior 11.6 mm, medial–lateral ± 1 mm, depth from dural surface 6 mm. Each of the 4 brainstem sites had a total of 0.5 µL BDA injected at a rate of 0.1 µL every 30 s, and the needle remained in place for 1 min after the final injection to ensure proper diffusion into the tissue. To allow for axonal transport of the anterograde tracer, the rats remained alive for an additional 3 weeks post‐injection. They then received a lethal dose of isoflurane, were transcardially perfused with 0.9% saline followed by 4% paraformaldehyde (PFA; Sigma 441 244) in 0.1 m PBS, and the spinal cords were harvested.

### Immunocytochemistry and Immunohistochemistry

Fixed cells from the reactive astrogliosis model were permeabilized with 0.1% Triton X‐100 in PBS for 5 min, blocked with 1% BSA in PBS for 30 min, and stained with primary antibodies (dilutions made in 1% BSA in PBS) for 1 h at room temperature. Samples were washed three times with 0.05% Tween‐20 in PBS and stained with secondary antibodies (dilutions made in PBS) for 1 h at room temperature or overnight at 4 °C. The primary antibodies and dilutions used were: chicken anti‐GFAP (AbCam ab24674 1:100), mouse anti chondroitin sulfate (Sigma Aldrich C8035 1:100), rabbit anti‐ß3‐tubulin (Cell Signaling Technologies 5666S 1:300). The secondary antibodies and dilutions: goat anti‐chicken 488 (A‐21449), goat anti‐mouse 590 (A‐11004), and goat anti‐rabbit 647 (A‐21245) (all Thermo Fisher 1:400). Nuclei were counterstained with DAPI. Fluorescence was imaged with a Nikon Ti Eclipse microscope was equipped with filters for FITC, Texas Red and DAPI, and Cy5.

BSA‐FITC‐laden MCMs were delivered to the spinal cord of 3 animals, as described above in 5.10, except the injections were done immediately after the contusion. The animals were euthanized 6 h post‐injection and the spinal cord tissue was collected. Transverse cross‐sections of the tissue at the injury/injection site and 4 mm caudal and rostral to the injury site were cut and the tissue was examined directly for green fluorescence via a Nikon Eclipse inverted fluorescence microscope using a FITC filter cube. The data presented are representative images.

CM‐mßGal‐laden MCMs were delivered to the spinal cord of 3 animals, as described above in 5.10. 12 h post‐delivery, the animals were euthanized. Transverse cross‐sections of the tissue at the injury/injection site and 4 mm caudal and rostral to the injury site were cut, and the tissues were stained to detect the presence of transgenically expressed *lacZ* using the *β*‐Gal Staining Kit (ThermoFisher) according to the manufacturer's instructions. The tissue was examined directly for blue‐purple staining indicating the presence of *lacZ* via a Nikon Eclipse inverted microscope on brightfield and color Nikon camera. The data presented are representative images.

CM‐mEGFP‐laden MCMs were delivered to the spinal cord of 3 animals as described in 5.10. 12 h post‐delivery, the animals were euthanized, the spinal cords removed, and the tissue fixed in the 4% paraformaldehyde overnight at 4 °C. Whole tissues were permeablized in 0.5% Triton‐X in PBS for 30 min 3× at RT and then incubated in blocking buffer (0.5% Triton‐X, 10% bovine serum albumin, and 0.2% sodium azide in PBS) overnight at 4 °C. The blocked tissues were incubated in primary antibodies rabbit anti‐GFP (Abcam ab6556 at 1:500) mouse anti‐chondroitin sulfate (Sigma Aldrich C8035 at 1:100) diluted in blocking buffer for 4 days at 4 °C. Tissues were washed in blocking buffer 3× for 1 day each at 4 °C. Washed tissues were incubated in secondary antibodies goat anti‐rabbit 488 (ThermoFisher A‐27034 at 1:500) and goat anti‐mouse 590 (ThermoFisher A‐11004 at 1:500) diluted in blocking buffer. Nuclei were counterstained with DAPI. Tissues were again washed in blocking buffer 3× for 1 day each at 4 °C. The stained tissues were then serially dehydrated in increasing amounts of methanol in PBS (25%, 50%, 75%, and 100% methanol), with each incubation occurring at 4 °C for 24 h. After equilibration in 100% methanol, the samples were cleared by incubating in a 1:2 mixture of benzyl alcohol and benzyl butyrate for 48 h at 4 °C. The cleared and stained tissues were imaged in serial *X*, *Y* and *Z* planes on a Zeiss AiryScan laser‐scanning confocal microscope with 350, 488 and 594 lasers, and 3D reconstructions were created in the Imaris image analysis suite. The data presented are representative images.

6 weeks after injury, the rats were perfused transcardially with 0.9% saline followed by 4% PFA in 0.1 m PBS, and 20 mm of spinal cord containing the injury site were harvested. The spinal cords of 4–5 animals per treatment group were collected for immunohistochemistry and submerged in 4% PFA for 24 h and then submerged in 30% sucrose in 0.1 m PBS for 48 h, all at 4 °C. After fixation, the spinal cord segments were cut sagittally along the ventral split, frozen in Tissue‐Tek and cut 30 µm thick. The slides were washed with 0.1 m PBS, followed by 1 h in blocker (4% normal donkey serum, 1% BSA, 0.3% Triton, 0.1 m PBS). The slides were submerged in the primary solution, consisting of 1:1000 rabbit‐anti‐GFAP (Abcam ab7260) to label astrocytes, combined with either 1:200 mouse anti‐chondroitin sulfate (CS‐56; SIGMA C8035) to label CSPGs or 1:100 mouse anti‐unsulfated chondroitin (1B5; MBDbioproducts 1 042 014) to label the unsaturated disaccharide of unsulfated chondroitin generated by ChABC digestion, in 0.1 m PBS with 1% BSA, for 24 h at 4 °C. The slides were then incubated for 1 h at room temperature in the secondary solution consisting of 1:500 donkey‐anti‐rabbit 594 (Invitrogen A21207) and 1:500 donkey‐anti‐mouse 488 (Invitrogen A21202) in 0.1 m PBS with 1% BSA. After IHC, a 10× merged image of the entire spinal cord section was taken with a Keyence BZ‐9000 microscope using the same microscope parameters for all sections imaged. ImageJ was used to measure the percent of the area labeled positive for CSPGs or digested CSPGs. For this analysis, 3 mm of spinal cord centered on the injury was outlined along the dura mater, and the percent area labeled positive for CSPGs, or digested CSPGs was quantified using the same threshold for all sections. The results for each rat are the average of 3 sections analyzed for CSPGs and 3 sections analyzed for digested CSPGs. Infarct size was also measured by outlining the cystic cavity within the GFAP labeled sections using ImageJ.

To quantify the percent of BDA‐labeled axons that extended through the injured spinal cord, BDA labeled axons were counted on transverse sections rostral and caudal to the injury in 4–5 animals from each treatment group not sacrificed for immunohistochemistry analysis of proteoglycan content. A 3 mm spinal cord segment 9 mm rostral to the injury site and a 3 mm segment of spinal cord 11 mm caudal to the injury site were submerged in 4% PFA for 24 h and then placed into 30% sucrose in 0.1 m PBS for 48 h. The spinal cord segments were frozen in Tissue‐Tek and sectioned transversely at a thickness of 20 µm. The slides were rinsed in 0.1 m PBS and blocked for 1 h (4% normal donkey serum, 1% BSA, 0.5% Triton, in 0.1 m PBS). The slides were then submerged in a 1:500 ratio of Streptavidin Alexa Fluor 594 conjugate (Thermo Fisher S‐11227) in 0.1 m PBS for 2 h in the dark at room temperature, and then rinsed and coverslipped. The number of axons in the white matter with a diameter of 1 µm or larger were counted on 3 transverse sections rostral and caudal to the injury, and then the percent of axons extending through the injured spinal cord was calculated using the average of the 3 caudal sections divided by the average of the 3 rostral sections.

To assess differences in axonal growth near the lesion, 5‐HT positive axons were identified in the 4–5 animals from each treatment group sacrificed for immunohistochemistry by immunolabeling of the plasma membrane 5‐HT transporter.(Belmer 2019) Sagittal frozen sections were cut 30 µm thick, washed with 0.1 m PBS, and placed in a blocking solution for 1 h (4% normal goat serum, 1% BSA, 0.3% Triton, 0.1 m PBS). The slides were submerged in the primary solution, consisting of 1:1000 chicken‐anti‐GFAP (Abcam ab4674) to label astrocytes and 1:450 rabbit anti‐Serotonin (5‐HT) Transporter (5‐HTT; Millipore 602–622) to label 5‐HT axons, in 0.1 m PBS with 1% BSA, for 24 h at 4 °C. After rinsing, the slides were then incubated for 1 h at room temperature in the secondary solution consisting of 1:500 goat‐anti‐chicken 594 (ThermoFisher A‐11042) and 1:500 goat‐anti‐rabbit 488 (ThermoFisher A‐11008) in 0.1 m PBS with 1% BSA. For quantifying axonal growth, a 40× image was taken directly rostral to the injury with a Keyence BZ‐9000 microscope using the same microscope parameters for all sections imaged. Then ImageJ was used to measure the percent of the area labeled positive for 5‐HT axons using the same threshold for all images.

### Animal Study Oversight

All animal studies were performed in accordance with the United States National Institutes of Health Guidelines for Animal Care (IACUC, protocol# M005958), and all procedures for animal experiments and care were approved by The University of Wisconsin‐Madison Institutional Animal Care and Use Committee.

### Figure Generation and Statistics

All schematic graphics were created by the author using Adobe Illustrator and Photoshop except for the microparticle, which was commissioned from a professional graphic designer.

### Data Analysis and Statistics

Data analysis, statistics, and graphs were generated in GraphPad Prism. The number of animals per treatment group, statistical and post hoc tests, and degree of significance for each comparison are denoted in each figure caption. For each analysis of experimental results from our SCI injury study, the results presented are after the data was parsed for outliers using the nonlinear regression “Robust regression and Outlier removal” (ROUT) in Prism with the default Q coefficient of 1%.^[^ ^86^ ^]^

## Conflict of Interest

W.L.M. is a co‐founder and Chief Science Officer at Stem Pharm, Inc. and Dianomi Therapeutics. A.S.K. is a consultant to Dianomi Therapeutics.

## Supporting information

Supporting Information

Supplemental Video 1

Supplemental Video 2

## Data Availability

The data that support the findings of this study are available from the corresponding author upon reasonable request.
