# Supplementary material for: A Localized Materials‐Based Strategy to Non‐Virally Deliver Chondroitinase ABC mRNA Improves Hindlimb Function in a Rat Spinal Cord Injury Model
Source: Adv Healthc Mater. 2022 Aug 25;11(19):2200206. doi: 10.1002/adhm.202200206 (PMC10031873; doi:10.1002/adhm.202200206)
Supplement: Supplementary file 1 — Supporting Information [file ADHM-11-2200206-s003.pdf]

ADVANCED HEALTHCARE MATERIALS

Supporting Information

for *Adv. Healthcare Mater.*, DOI 10.1002/adhm.202200206

A Localized Materials-Based Strategy to Non-Virally Deliver Chondroitinase ABC mRNA Improves Hindlimb Function in a Rat Spinal Cord Injury Model

Andrew S. Khalil, Daniel Hellenbrand, Kaitlyn Reichl, Jennifer Umhoefer, Mallory Filipp, Joshua Choe, Amgad Hanna and William L. Murphy**

Supplementary Figures

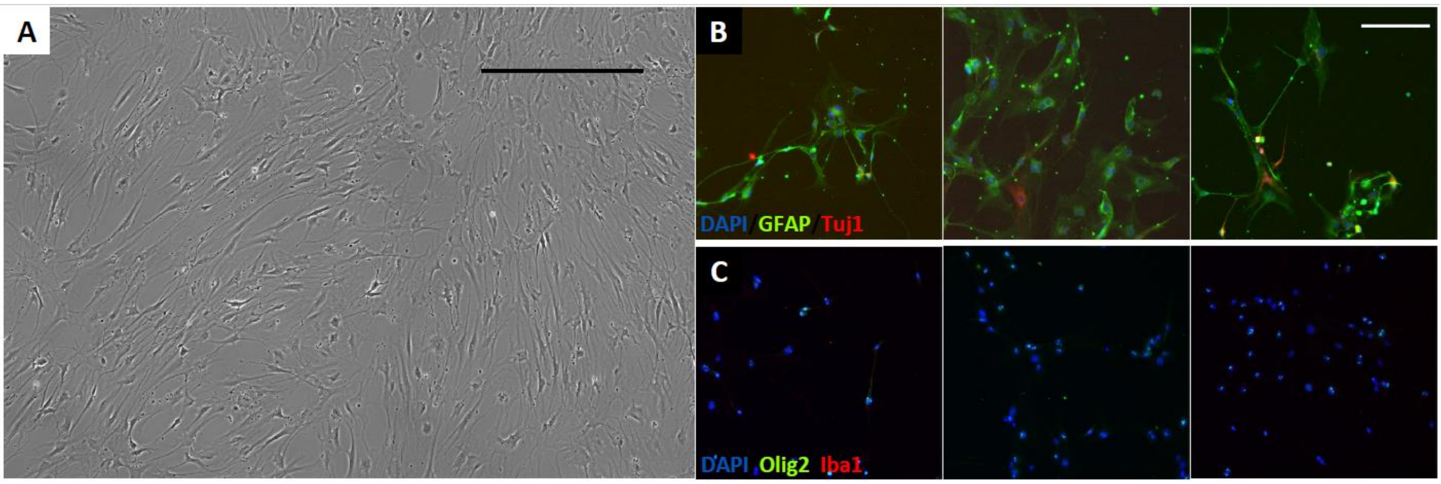

Supplementary figure 1. Characterization of primary glial cells isolated from rat spine. (A) Phase image of glial cells after isolation, expansion, cryopreservation, and thaw. scale bar = 500 µm (B) Three representative immunofluorescence images, including the cropped image from panel 1C, for astrocyte (GFAP) and neuronal (Tuj1) subpopulations within the primary spinal cells. (C) Three representative immunofluorescence images, including the cropped image from panel 1C, for oligodendrocyte (Olig2) and microglia (Iba1) subpopulations within the primary spinal cells. scale bar for B & C = 200 µm

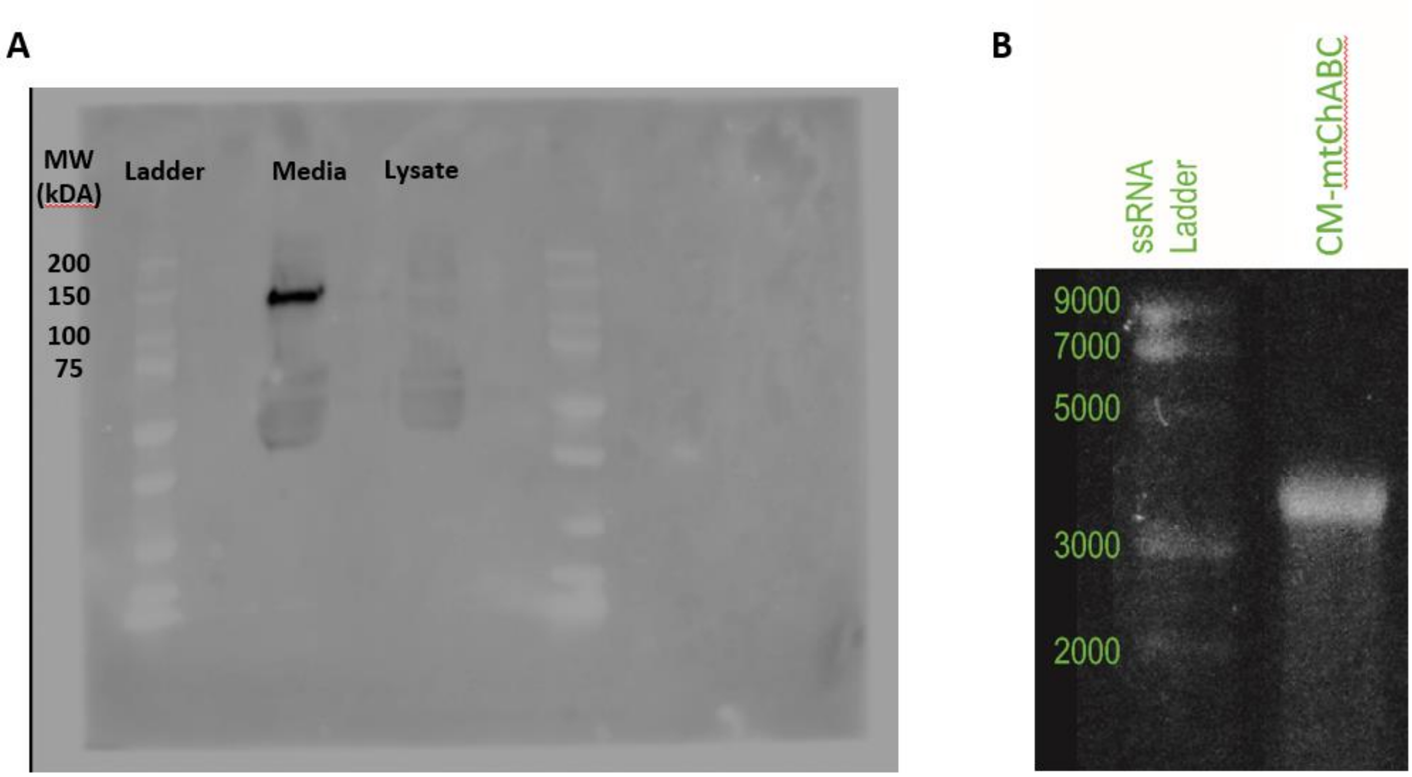

Supplementary figure 2. Characterization of eukaryotic mtChABC expression and in vitro transcription of CM-mtChABC mRNA. (1) Uncropped 10% polyacrylamide gel from figure 2B. (B) 1.5% agarose electrophoresis gel of denatured CM-mtChABC synthesis measured against denatured single-strand RNA ladder of known size.

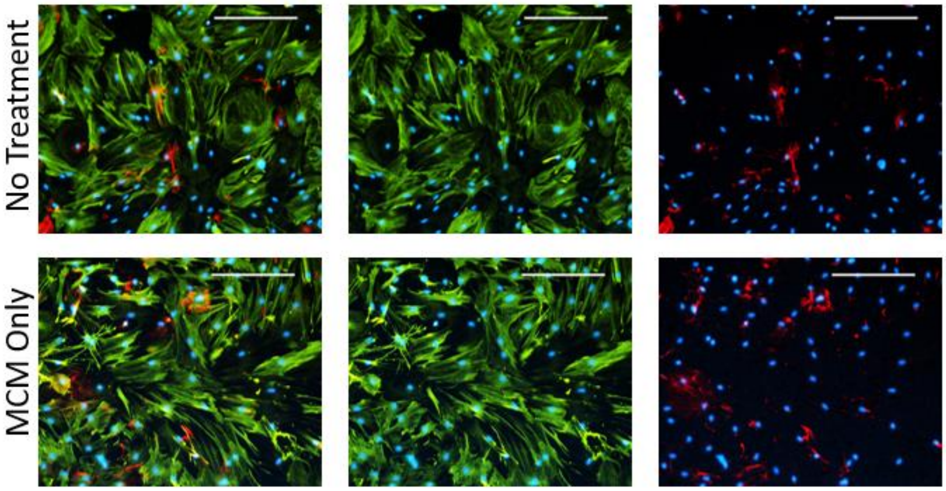

Supplementary figure 3: MCM in vitro astrogliosis control. Representative epifluorescence micrograph of untreated and MCM-only vehicle-treated astrocytes in the astrogliosis model show no differences CSPG reduction with nuclei in blue, GFAP in green, and CSPG in red. Scale bar = 50 µm

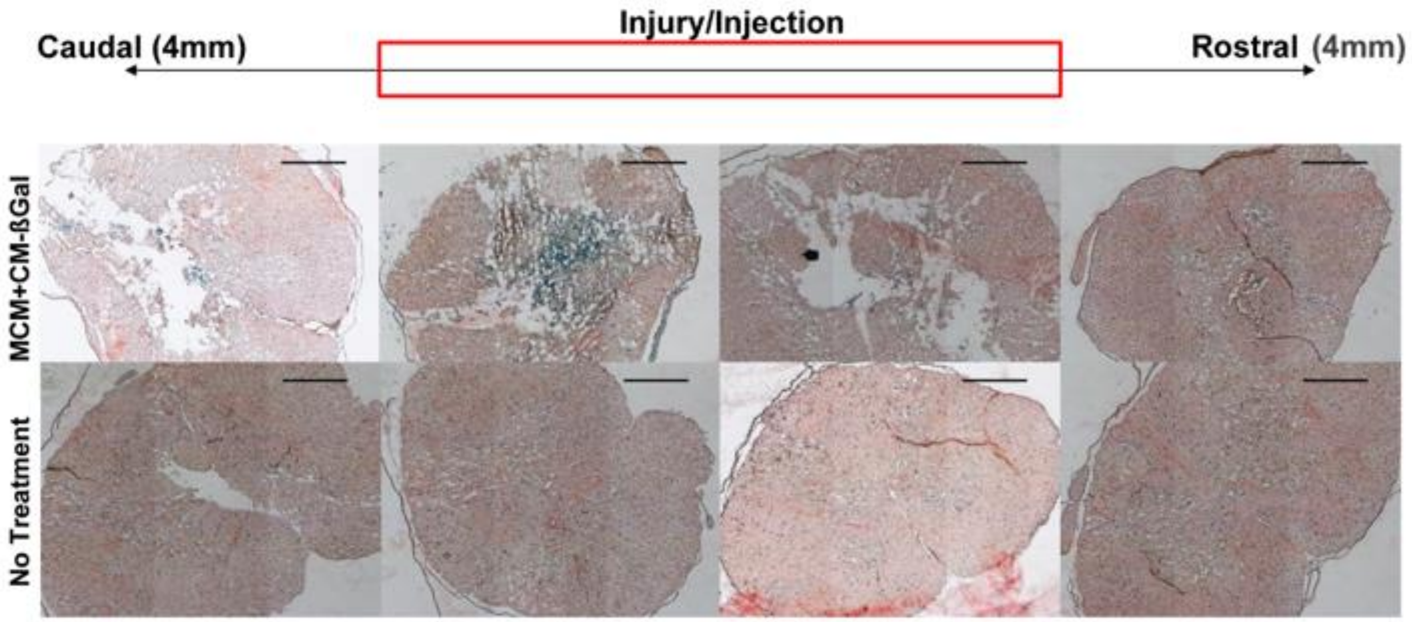

Supplementary figure 4. MCM-mediated mRNA delivery via stereotactic direct injection results in localized transgene expression of active enzyme in the injured spinal cord. Representative histology of CM-mßGal-laden MCMs delivered to contused spinal cord one week post-injury show positive staining for ßGal activity (blue) only at the injection site, indicating successful local transfection of the glial scar. Minimal or no ßGal staining was observed 4mm caudal and rostral of the injection. Scale bar = 500 µm

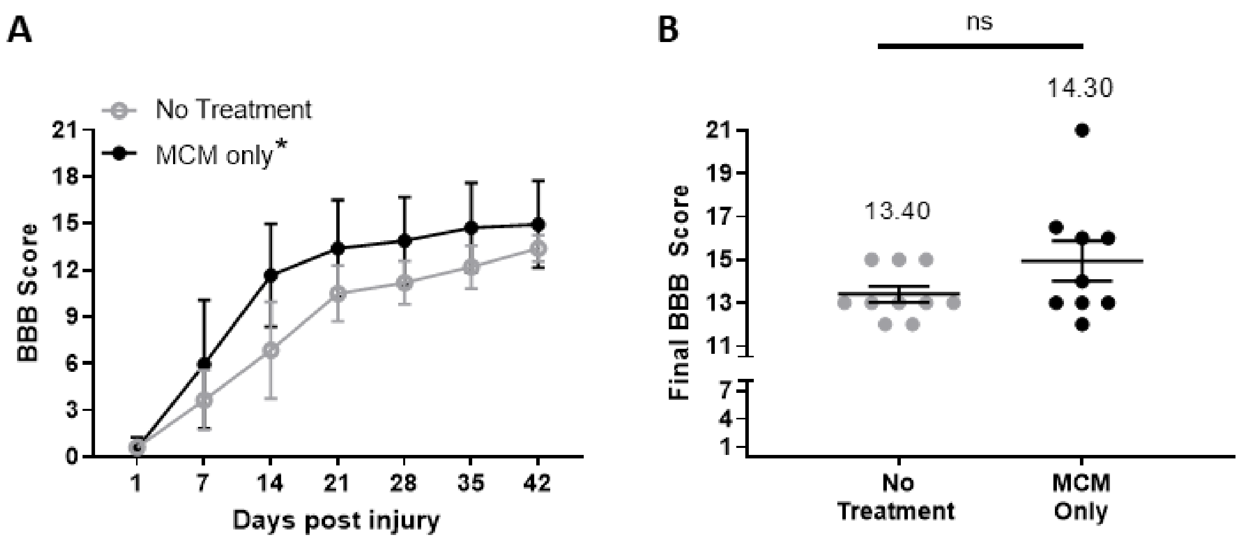

Supplementary figure 5: MCM vehicle control. (A) BBB scoring of rats with SCI vs. time with rats scored once per week for the no treatment and MCM-only vehicle controls. Mean+95% CI N=10 * p-value < 0.05 respectively, by two-way ANOVA with Dunnett's post hoc analysis relative to no treatment control. (B) No treatment and vehicle controls final BBB scores did not show a significant improvement relative to the no treatment control. Mean+95% CI N=10.

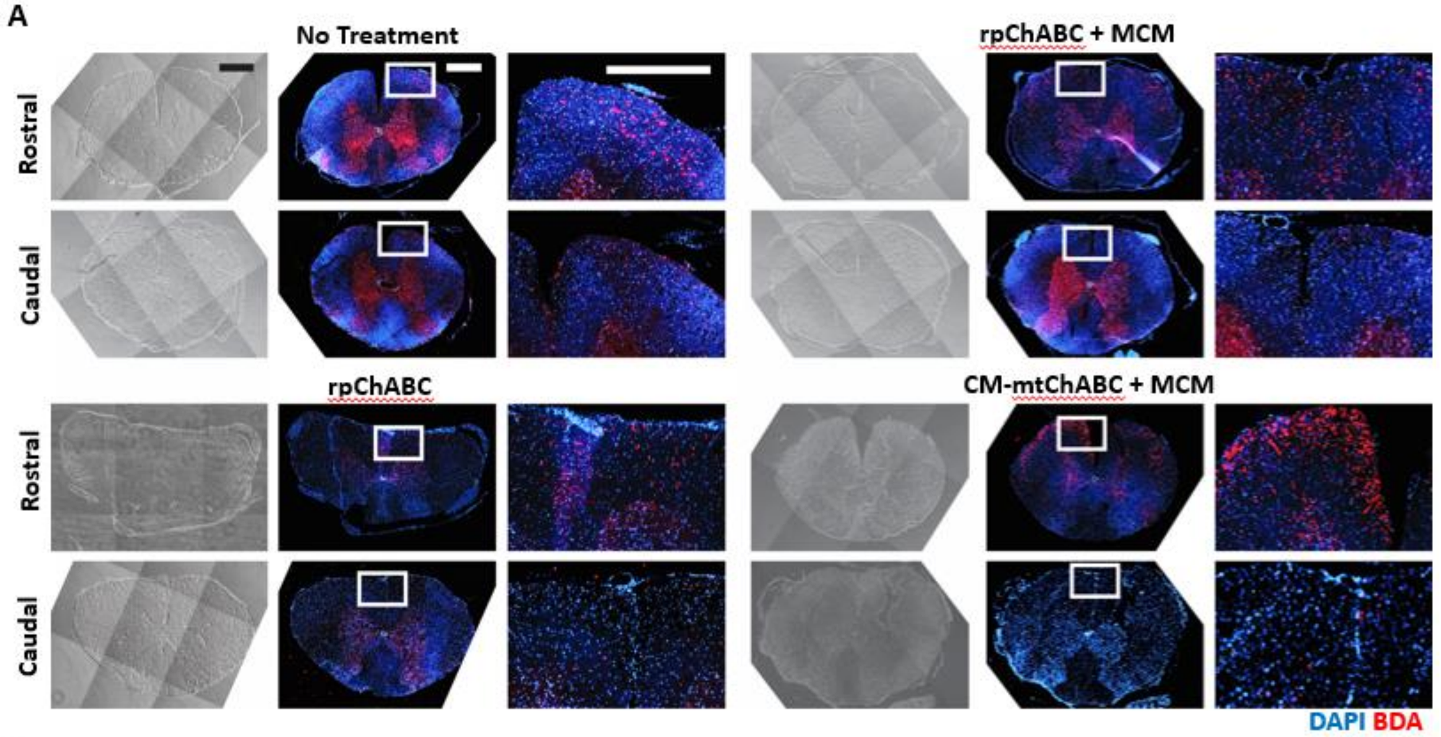

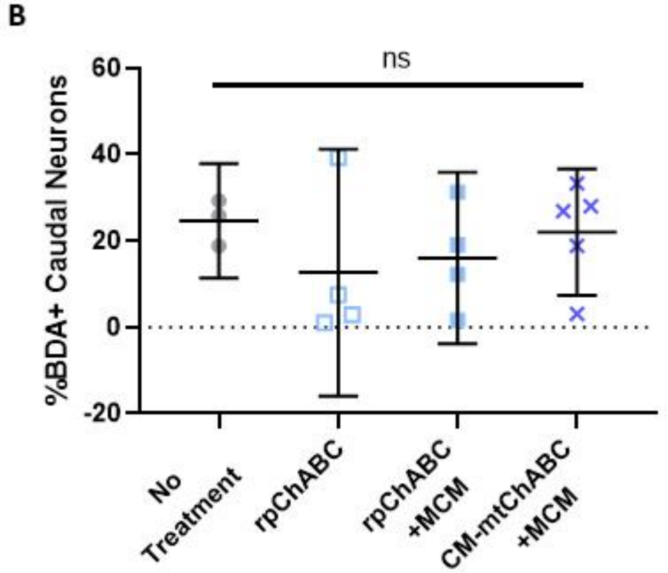

Supplementary figure 6: Analysis of axon tracing. (A) Representative images of transverse sections 9 mm rostral and 11 mm caudal to the injury site with streptavidin staining of BDA injections into the RN and RF. scale bars = 500 µm (B) Traced axons from BDA injections represented as a percentage of labeled axons, caudal to the injury site out of the number of labeled axons rostral to the injury site. N = 3-5 ns p-value > 0.05 by one-way ANOVA with Dunnett's post hoc analysis relative to no treatment control

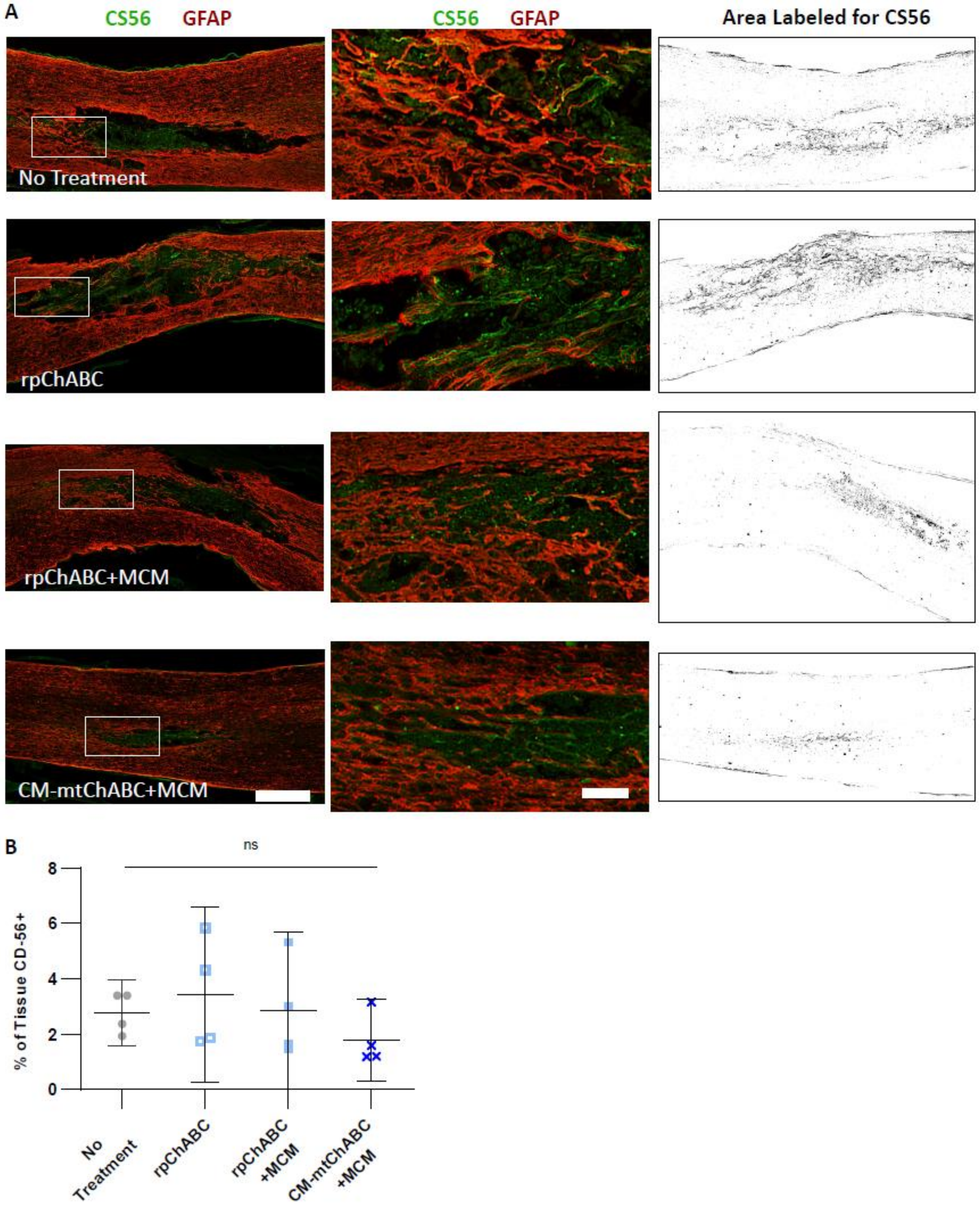

Supplementary figure 7. CSPG and GFAP SCI immunohistochemistry and analysis. (A) Representative images showing sfull-length CS56 positive CSPGs (green) and astrocytes labeled with GFAP (red), high magnification inset areas from the white

outlined region, and representative automated analysis threshold images of CS56 positive area. (B) The percent of CS56 positive area was not significantly different among groups. Mean+95% CI, N=4, scalebars = 500 µm on whole section images and 50 µm on high magnification images.

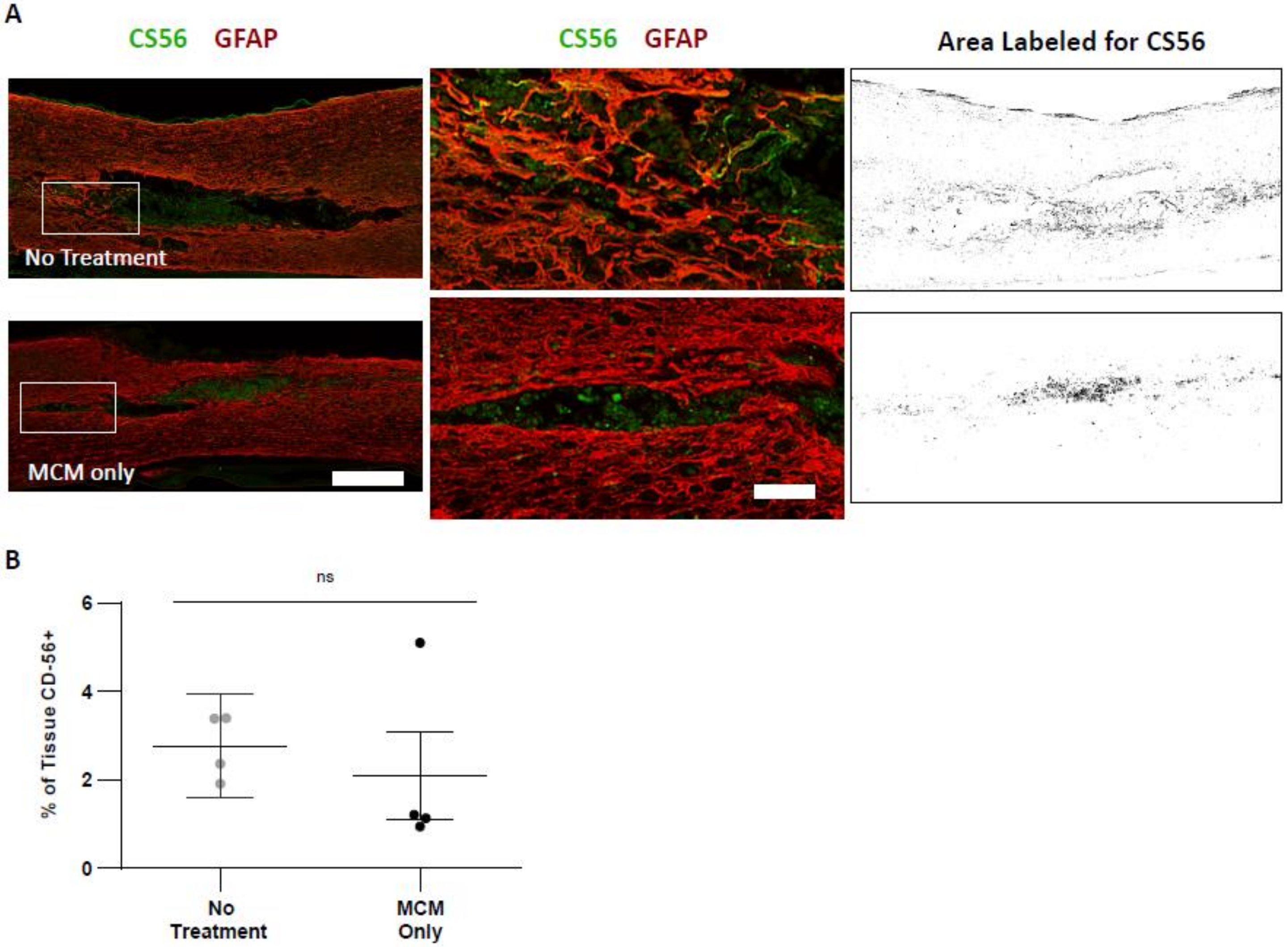

Supplementary figure 8. Intact SPG and GFAP SCI immunohistochemistry and analysis for vehicle control. (A) Representative vehicle images showing digested CSPGs labeled with 1B-5 (green), counterstained for astrocytes with GFAP (red), high magnification inset areas from the white outlined region, and representative automated analysis threshold images of 1B-5 positive area. (B) Immunohistochemical analysis of degraded chondroitin sulfate products (1B-5). Mean+95% CI, N=4, scalebars = 500 µm on whole section images and 50 µm on high magnification images.

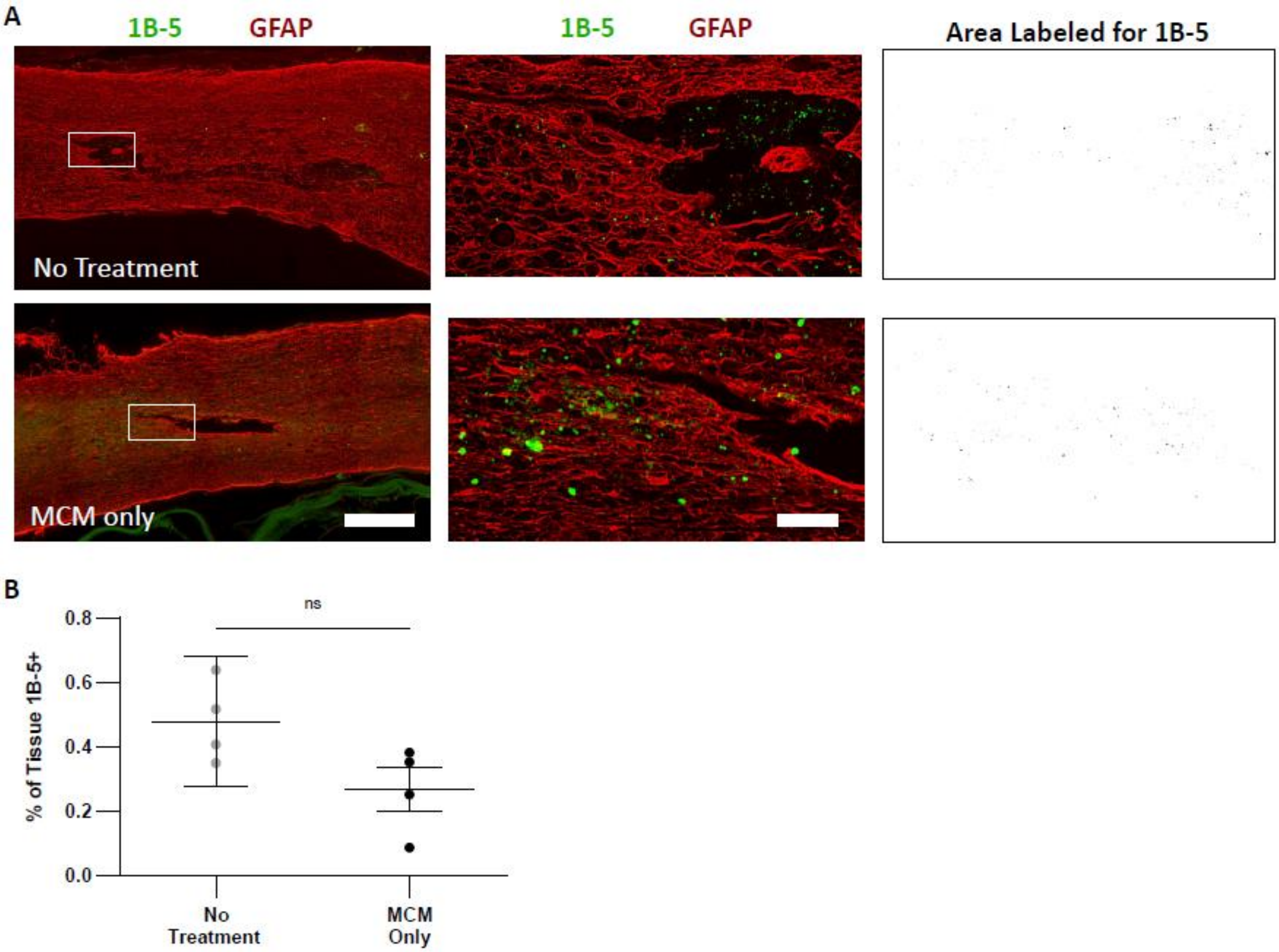

Supplementary figure 9. Degraded CSPG and GFAP SCI immunohistochemistry and analysis for vehicle control. (A) Representative vehicle images showing digested CSPGs labeled with 1B-5 (green), counterstained for astrocytes with GFAP (red), high magnification inset areas from the white outlined region, and representative automated analysis threshold images of 1B-5 positive area. (B) Immunohistochemical analysis of degraded chondroitin sulfate products (1B-5). Mean+95% CI, N=4, scalebars = 500 µm on whole section images and 50 µm on high magnification images.

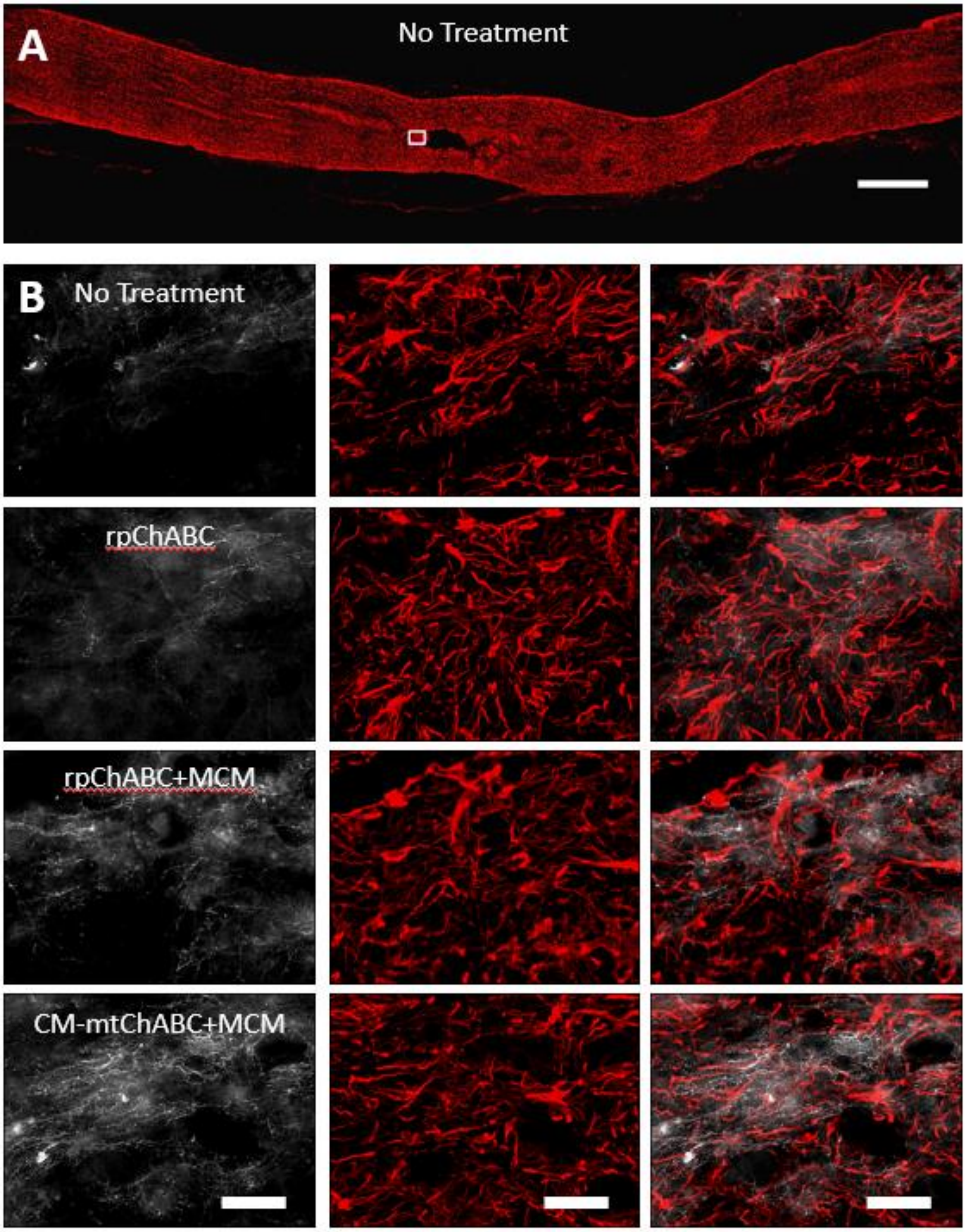

Supplementary figure 11. Low magnification 5-HT representation and GFAP counterstain. **Supplementary figure 11. Low magnification 5-HT representation and GFAP counterstain.** (A) Sagittal section displaying where the high magnification micrographs (box) were taken rostral to the injury for analysis. (B) Representative images showing 5-HT positive serotonergic axons sprouting (white), counterstained for astrocytes with GFAP (red). A = 500 µm and B = 50 µm.

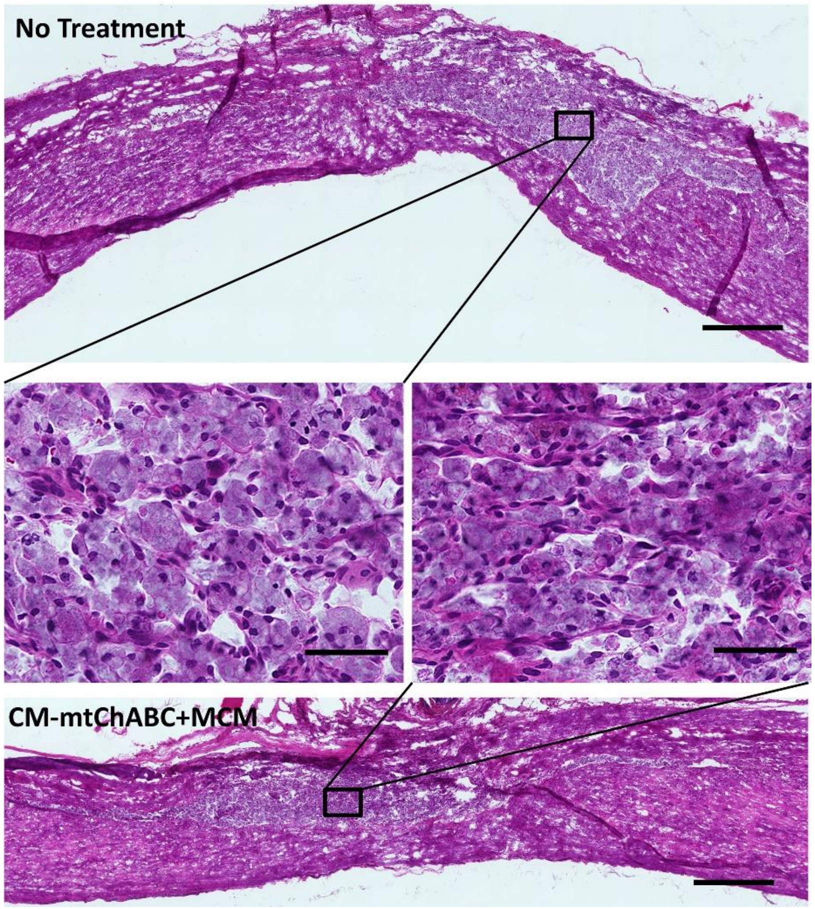

Supplementary figure 11. Analysis of immune cells with H&E staining on sagittal sections. The epicenter of the injury contained dense populations of ameboid macrophages, indicating chronic inflammation. There were no observed differences in macrophage infiltration between No Treatment (Top) and CM-mtChABC+MCM (bottom). Scalebars: high magnification images = 50 µm and low magnification images = 500 µm.

Supplementary movie 1: Hindlimb locomotion six weeks post-injury in representative no treatment saline control animal

Supplementary movie 2: Hindlimb locomotion six weeks post-injury in representative CM-mtChABC+MCM treatment animal
